# Discontinuation of immune checkpoint inhibitors for reasons other than disease progression and the impact on relapse and survival of advanced melanoma patients. A systematic review and meta-analysis

**DOI:** 10.3389/fimmu.2025.1524945

**Published:** 2025-01-31

**Authors:** Konstantinos Lallas, Eftychia Chatziioannou, Derya Durak, Georg Frey, Lina Maria Serna-Higuita, Marie-Lena Rasch, Athanassios Kyrgidis, Eleni Timotheadou, Zoe Apalla, Ulrike Leiter, Lukas Flatz, Aimilios Lallas, Teresa Amaral

**Affiliations:** ^1^ Department of Medical Oncology, School of Medicine, Aristotle University of Thessaloniki, Thessaloniki, Greece; ^2^ Center for Dermato-oncology, Department of Dermatology, Eberhard Karls University of Tübingen, Tübingen, Germany; ^3^ Clinical Epidemiology and Applied Biostatistics, Eberhard Karls University of Tübingen, Tübingen, Germany; ^4^ Oral and Maxillofacial Surgery, Aristotle University of Thessaloniki, Thessaloniki, Greece; ^5^ Second Department of Dermatology, School of Medicine, Aristotle University of Thessaloniki, Thessaloniki, Greece; ^6^ Cluster of Excellence iFIT (EXC 2180) “Image-Guided and Functionally Instructed Tumor Therapies”, Tübingen, Germany; ^7^ First Department of Dermatology, School of Medicine, Aristotle University of Thessaloniki, Thessaloniki, Greece

**Keywords:** immunotherapy, immune checkpoint inhibitors, therapy discontinuation, stage IV, overall survival, melanoma

## Abstract

**Background:**

Despite durable responses achieved with Immune Checkpoint Inhibitors (ICIs), data about optimal duration of treatment, especially in the context of adverse events, remain scarce.

**Objective:**

To systematically review the evidence concerning the impact of treatment discontinuation with ICIs for reasons other than progressive disease (PD) on relapse rates and survival of melanoma patients.

**Methods:**

A systematic literature search was conducted in three electronic databases until July 2024. Studies referring to melanoma patients who ceased ICIs electively (i.e. due to complete response (CR), protocol completion or patient/physician’s wish) or due to treatment-limiting toxicities (TLTs) were selected. Relapse rates (RRs) post cessation, time to PD, rechallenge and disease control rate (DCR) after 2^nd^ course were the main outcomes. Random-effects models were preferred, and subgroup and sensitivity analyses were conducted to investigate possible sources of heterogeneity.

**Results:**

38 and 35 studies were included in qualitative and quantitative synthesis, respectively. From 2542 patients discontinued treatment with ICIs electively or due to TLTs, 495 experienced progression [number of studies (n)=34, RR 20.9%, 95%CI 17.1 – 24.7%, I^2^ 85%) and higher rates were detected in patients with TLTs compared to elective discontinuation. Mean time to PD was 14.26 months (n=18, mean time 14.26, 95%CI 11.54 – 16.98, I^2^ 93%) and was numerically higher in patients who ceased for CR compared to patients with TLTs. Treatment duration before cessation was not associated with risk and time to relapse, while mucosal melanomas and non-CR as BOR during treatment led to increased risk for relapse and shorter time to PD compared to other histologic subtypes or CR. Rechallenge with ICI resulted in 57.3% DCR and 28.6% pooled CR rates (n=22, CR rate 28.6%, 95%CI 17.1 – 40.2, I^2^ 68%). Heterogeneity among studies was high, but subgroup analysis based on type of ICI used (anti-CTL4 and anti-PD1 inhibitor or anti-PD1 monotherapy) and type of study (RCTs or observational studies), along with sensitivity analyses did not reveal significant alterations in results.

**Conclusion:**

Discontinuation of ICIs in patients without progression is possible. Outcomes to rechallenge with ICIs may differ depending on the reason for discontinuation, but remains a considerable option.

**Systematic review registration:**

https://www.crd.york.ac.uk/prospero/, identifier CRD42024547792.

## 1 Introduction

Immune checkpoint inhibitors (ICIs) revolutionized the treatment of metastatic melanoma patients, contributing to significantly improved overall survival (OS) rates compared to other treatment modalities (1, 2). Importantly, responses on ICIs are considered durable and patients achieving complete or partial responses seem to remain on response, even after treatment cessation (3, 4). However, the optimal treatment duration for patients with melanoma without progression remains unknown (5), while the total duration of therapy with ICIs are determined arbitrary in treatment protocols, ranging from 2 years in some studies to treatment until progressive disease (PD) in others (1, 2, 6).

Careful consideration of treatment duration with ICIs lies in balancing response preservation after ICI cessation and toxicity avoidance, while reducing costs. Immune related adverse events (irAEs), and especially late-onset irAEs, remain a major issue, affecting quality of life of patients receiving ICI, which is particularly important in the context of complete response (CR) (7, 8). To answer this question, observational studies analyzed the impact of treatment discontinuation electively or due to AEs on relapse, but their results remain inconsistent (9–12). In addition, meta-analyses examining optimal duration of ICIs in solid tumors, including melanoma, did not report either a survival benefit of patients treated with fixed duration compared to treatment until disease progression, or explored factors influencing relapse risk comprehensively (13, 14).

Consequently, the main aim of our review was to systematically review all available evidence on disease relapse following ICI discontinuation for reasons other than progressive disease (PD), to evaluate the role of various factors, such as type of ICI or reason for discontinuation, in an extensive way, and to report post-relapse management of patients who experienced PD after ICI cessation.

## 2 Materials and methods

### 2.1 Guidelines followed

**Figure 1:**
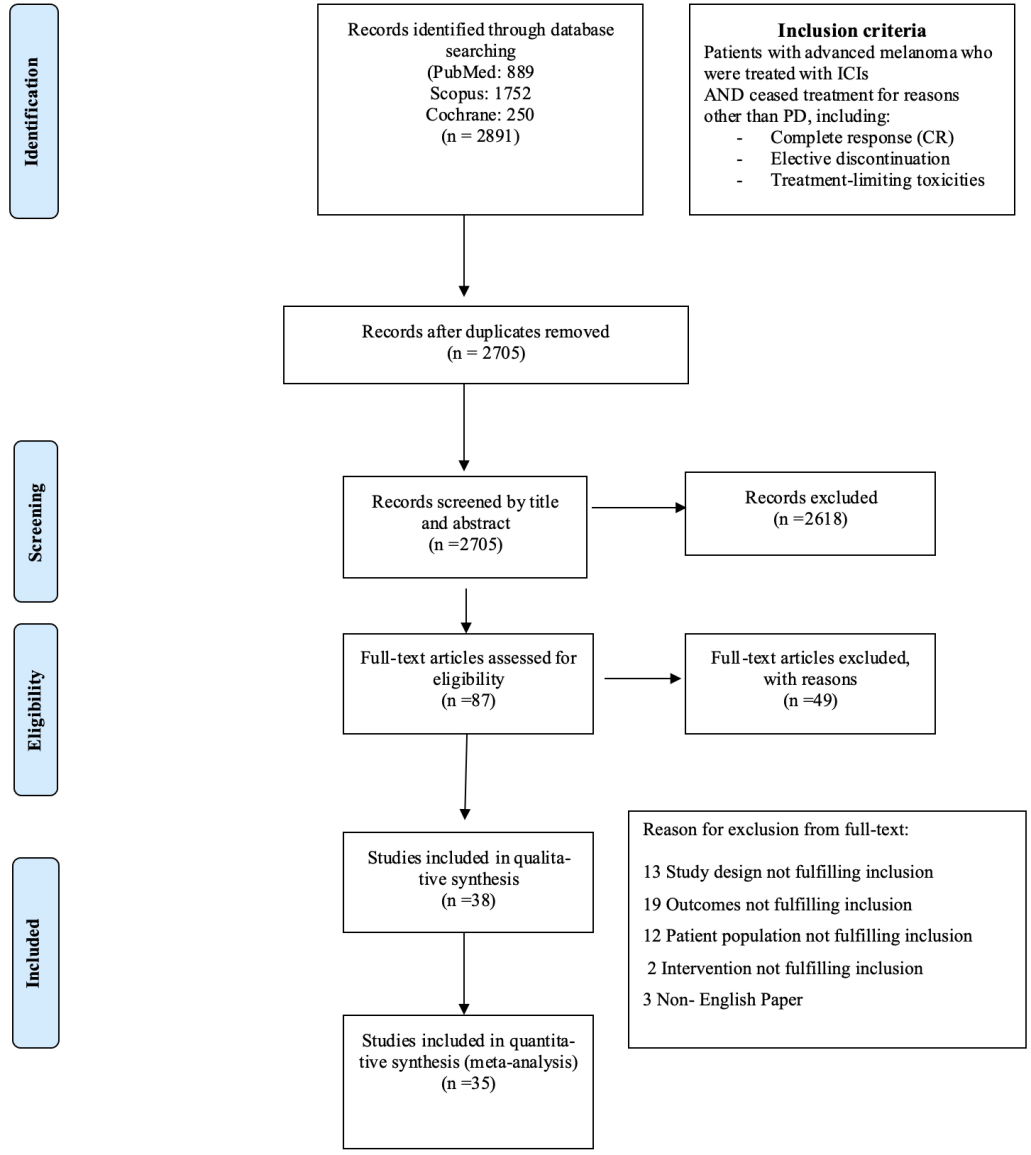
Flow chart diagram.

This systematic review followed the guidelines outlined in MOOSE (15) and PRISMA guidelines (16), where feasible. **Figure 1** displays the flow chart diagram. Study has been registered in PROSPERO (International Prospective Register of Systematic Reviews, PROSPERO ID: CRD42024547792).

### 2.2 Search strategy

To identify eligible studies, 4 independent investigators (KL, DD, GF, MLR) conducted thorough literature search in the following electronic databases: MEDLINE (PubMed), Scopus and Cochrane (CENTRAL) until July 2024 and arising discrepancies were resolved by a fifth investigator (TA). In addition, conferences and grey literature were screened and manual search of references of included studies was conducted to search for relevant studies. To obtain missing data for included studies, authors were contacted via email. A representative example of search string in PubMed is: (“immunotherapy”[MeSH Terms] OR “immunotherapy”[Title/Abstract]

OR “immune checkpoint inhibit*”[Title/Abstract] OR “ICI”[Title/Abstract] OR “ICB”[Title/Abstract] OR “anti-PD1”[Title/Abstract] OR “anti-PDL1”[Title/Abstract] OR “anti-CTL4”[Title/Abstract]) AND (“discontinue*”[Title/Abstract] OR “disconti*”[Text Word] OR “cessa*”[Title/Abstract] OR “stop*”[Title/Abstract] OR “premature disconti*”[Title/Abstract] OR “early disconti*”[Title/Abstract] OR “interrupt*”[Title/Abstract] OR “break”[Title/Abstract]) AND (“melanoma”[MeSH Terms] OR “melanoma”[Title/Abstract] OR “skin cancer”[Title/Abstract] OR “cutaneous melanoma”[Title/Abstract] OR (“melanoma”[MeSH Terms] OR “melanoma”[All Fields] OR “melanomas”[All Fields] OR “melanoma s”[All Fields]) and further analysis can be found in **Supplementary Marerial.**

### 2.3 Study selection

The following parameters were set as inclusion criteria: studies including patients with melanoma who were treated with immune checkpoint inhibitors (either combination anti-CTL4 and anti-PD1, or anti-PD1 monotherapy) for advanced disease, and who ceased treatment for reasons other than PD. Discontinuation after CR (verified by biopsy or per RECIST criteria), protocol completion and patient/physician choice (both defined as “elective discontinuation”), or due to the development of treatment-limiting toxicities (TLTs) were the main reasons for treatment cessation. Both randomized controlled trials (RCTs) and observational studies (including prospective or retrospective studies, cohorts, case-control studies and case-series) were included. Exclusion criteria encompassed studies that referred to treatment discontinuation for PD, or not providing the reason for discontinuation in a clear way, studies including patients treated in the adjuvant setting or receive treatment other than ICIs, or patients with conjunctival or uveal melanomas, studies not providing extractable data, reviews, meta-analyses, case reports and non-English papers.

### 2.4 Data extraction

Four independent researchers (KL, DD, GF and MLR) extracted data from the eligible studies that met the inclusion criteria. A standardized form was used to record the following parameters: (i) first author, (ii) year of publication, (iii) country in which the study was conducted, (iv) study design, (v) duration of follow up, (vi) number of patients analyzed and number of patients who discontinued treatment, (vii) reasons for treatment cessation (i.e. elective or due to toxicities), (viii) outcomes reported in every study [i.e. relapse rates, time to relapse after treatment cessation, rechallenge (type of treatment used for rechallenge and disease control rate (DCR), PFS and OS).

The primary outcome was relapse rate after treatment discontinuation for any reason other than PD. Time to PD after treatment discontinuation, rechallenge and DCR after rechallenge, progression-free survival (PFS) (defined as the time from randomization for RCTs or start of treatment for observational studies to PD), OS (defined as the time from randomization for RCTs or start of treatment for observational studies to death or censoring) were set as secondary outcomes.

Different analyses based on reason for discontinuation [(i) elective cessation after disease control (including patients with CR, partial response or stable disease)) and without TLTs, (ii) elective cessation on CR and (iii) discontinuation due to TLTs without progression], along with subgroup analysis regarding type of ICI used (anti-CTL4 and anti-PD1 or anti-PD1 monotherapy) and type of studies (RCTs or observational studies) were also conducted.

### 2.5 Risk of bias and study quality assessment

For RCTs, the Cochrane Risk of Bias tool (RoB) (version 5.1.0) was selected to evaluate the quality of included studies (17). For observational studies, quality of selected studies was assessed by 4 independent reviewers (KL, DD, GF and MLR), using Newcastle-Ottawa scale (NOS) to evaluate risk of bias (18). This scale consists of the following categories: (i) participant selection, with a maximum rating of four stars, (ii) comparability of study groups, with a maximum of two stars, and (iii) assessment of outcome or exposure, with the highest rating reaching three stars. NOS and RoB results are demonstrated in **Supplementary Tables 1** and **Supplementary Tables 2**, respectively.

### 2.6 Statistical analysis

Relapse rates (RRs) were summarized using raw proportions and the generic inverse variance method. Risk of relapse after elective discontinuation or due to AE was expressed with Odds Ratios and 95% Confidence Intervals (CIs). Also, regarding time to PD after discontinuation, pseudo-individualized patient – data (IPDs) were extracted from Kaplan – Meier curves using methodology described by Tierney et al. (19), or from swimmer plots, according to Pala et al (13). Median times were converted into mean time to PD and standard errors based on methodology described by McGrath et al (20). Random effect models were used for data synthesis, in cases of high heterogeneity. I^2^ index was preferred for determining heterogeneity extent among studies, with values lower than 30% being considered as low heterogeneity, values ranging from 30% to 60% as moderate and values >60% as considerable heterogeneity. Subgroup analyses and sensitivity analyses were conducted to investigate possible sources of heterogeneity. Risk of publication bias was examined with Funnel plots and Egger’s test for small-study effects. All statistical tests were two-sided, p-value<0.05 was considered statistically significant and the analysis was conducted with Review Manager v.5.4.1 and IBM SPSS v29.

## 3 Results

### 3.1 Description of results from literature search

Until July 2024, 2,705 eligible studies were identified after duplicates removal, 87 of which were full-text assessed for eligibility. From those, 49 were excluded (for detailed reasons for exclusion please see **Supplementary Table 3**) and 38 and 35 were included in the qualitive and quantitative synthesis, respectively (**Figure 1**). Three studies were included in systematic review only, due to lack of extractable data available in the other publications (21–23). Overall, 34 studies investigated the relapse rates after discontinuation of ICI for reasons other than PD, 22 reported time to relapse after discontinuation, while 22 had available data concerning ICI rechallenge and DCR thereafter (**Table 1**).

**Table 1:** Baseline characteristics of studies included in systematic review/meta-analysis, reasons for ICI discontinuation, outcomes analyzed and significant results of each study.

	First Author, Year of Publication	Country	Type of study (RCT, observational cohort or cross-sectional)	Number of patients analyzed/Number of patients who discontinued treatment (Reason for discontinuation)	Type of treatment (line of treatment)/Regimens	Median follow up time from treatment start (mo)	Outcomes	Results	SR or MA	GRADE/NOS
1	Fletcher et al., 2024 (24)	Multicenter (18 centers from Europe and US, 2010 - 2019)	Retrospective observational study	286/54 (elective, TLT, patients’ preference)	Metastatic setting (prior lines)/anti-PD1 monotherapy (pembrolizumab or nivolumab)	NA	% relapse rate, PFS, OS	11% relapse rate in metastatic setting. No significant impact of treatment duration on PFS and OS.	MA	8
2	Tang et al., 2024 (25)	Multicenter (China)	Open-label, single – arm, phase II clinical trial	128 (*limited mucosal patients*)/13 (completion of 2 years of treatment)	Metastatic setting (prior lines)/anti-PD1 (toripalimab)	16.9 (range 0.9 – 70.1)	% relapse rate, time to PD, rechallenge	70% relapse rate after elective discontinuation with a median of 12.1 months. All patients with anti-PD1 rechallenge demonstrated DCR.	MA	~
3	Chatzioannou et al., 2023 (26)	Germany (Single center, Tubingen, 2014 – 2018)	Retrospective observational study	265/40 (CR)	Metastatic setting (1^st^ line)/combination ICI, anti-PD1 monotherapy	22 (IQR 17-24)	% relapse, rechallenge, Time to PD, PFS, OS	20% relapse rate in patients with CR within a median time 15.3 from discontinuation. Type of ICI not associated significantly with time to PD.	MA	7
4	Ochenduszko et al., 2023 (10)	Spain (Multicenter, 2015 - 2021)	Retrospective observational study	35/35 (CR)	Metastatic setting (mainly 1^st^ line treatment)/anti-PD1 monotherapy (nivolumab or pembrolizumab)	49.3 (95%CI 43.8 – 52.2)	% relapse, time to PD, PFS, OS	14.2% relapse rate in patients with CR within 25.9-month post-cessation. Antibiotics use was associated with shorted time to PD.	MA	9
5	Rubatto et al., 2023 (9)	Italy (Multicenter, 2012 - 2021)	Retrospective observational study	237/237 (CR, TLT, patients’ preference)	Metastatic setting (prior lines)/Anti-PD1 monotherapy (nivo or pembro)	NA	% relapse, rechallenge, time to PD after cessation	14.3% relapse rates within a median of 12 months from cessation. Mucosal subtype was associated with increased risk for relapse post-discontinuation.	MA	7
6	Sadrolashrafi et al., 2023 (28)	USA (Single center, Nevada, 2008 – 2021)*	Retrospective observational study	190/190 (CR)	Metastatic setting (1^st^ line)/combination ICI, anti-PD1 monotherapy	69.3	% relapse, time to PD, rechallenge, PFS, OS	1.9% relapse rate after cessation for CR. 70% DCR after rechallenge and a median OS not reached.	MA	7
7	Sharma et al., 2023 (29)	USA (Single center)	Retrospective observational study	44/11 with melanoma (disease control, TLTs)	Metastatic setting (NA)/anti-PD1 monotherapy	NA	% relapse, rechallenge	36% relapse rate after cessation with similar rates in patients with elective discontinuation or toxicities.	MA	6
8	Warburton et al., 2023 (48)	Australia Single center, Perth, 2013 – 2021)	Retrospective observational study (exploratory analysis)	34/34 (TLT, subgroup analysis for response to treatment)	Metastatic setting (prior lines)/combination ICI	NA	% relapse, rechallenge, PFS, OS	47% relapse rate after discontinuation for TLTs, PFS and OS were longer in patients who stopped treatment in CR compared to non-CR patients	MA	6
9	Dimitriou et al., 2022 (30)	Australia (MIA, 2013-2018)	Retrospective observational study	104/35 (response, TLT, patient choice)	Metastatic setting (prior lines)/combination ICI, anti-PD1 monotherapy	NA	% relapse	11% relapse rate, with similar percentages in patients who ceased treatment on CR or due to TLTs	MA	7
10	Ellebaek et al., 2022 (31)	Denmark (Cancer registry)	Retrospective observational study	140 (CR, PR patients)/140 (TLT or physician/patient choice)	Metastatic setting (mainly 1^st^ line)/combination ICI, anti-PD1 monotherapy	NA	%relapse, PFS, OS	20% relapse rate after discontinuation, and statistically significantly prolonged OS in patients who ceased electively compared to those due to TLTs	MA	8
11	Ferdinandus et al., 2022 (32)	Germany (Single center, Essen, 2010 – 2020)	Retrospective observational study	38/38 (disease control, separate analysis for toxicities or CR)	Metastatic setting (prior lines)/ combination ICI, anti-PD1 monotherapy	48.6	% relapse, time to PD, rechallenge, OS	15% relapse rate with shorter time to PD in patients with non-CMR in PET/CT. 75% DCR after ICI rechallenge.	MA	8
12	Kartolo et al., 2022 (27)	USA (Single center, 2014 – 2019)	Retrospective observational study	96/39 (toxicity or treatment protocol completion without PD)	Metastatic setting (prior lines)/ combination ICI, anti-PD1 monotherapy	NA	% relapse, [time to PD, OS (only subgroups)]	48% relapse rate post discontinuation, similar rates between elective or due to TLTs. Not significant difference in OS between elective discontinuation of due to TLTs	MA	6
13	Perez et al., 2022 (12)	USA (Single center, Nevada, 2015 – 2021)	Retrospective observational study	46/46 (CR)	Metastatic setting (1^st^ line)/combination ICI, anti-PD1 monotherapy	NA	% relapse, Time to PD, OS	8.6% relapse rate in patients with CR and a PD within 27.4 months post-discontinuation. OS NR in patients with CR	MA	7
14	Asher et al., 2021 (34)	Israel (Single center, 2014 – 2019)	Retrospective observational study	106/106 (disease control, separate analysis for toxicities or CR)	Metastatic setting (prior lines)/ combination ICI, anti-PD1 monotherapy	39.1	% relapse, time to PD, rechallenge, PFS, OS	32% relapse rate within a median 8.5 months post discontinuation. 68% DCR rate after ICI rechallenge. CR was associated with lower risk for progression and higher PFS rates compared to non-CR or steroids use.	MA	8
15	Dimitriou et al., 2021 (33)	Multicenter (France, Germany, Switzerland, Italy)	Retrospective observational study	125/125 (all CR, separate analysis for toxicities with CR)	Metastatic setting (prior lines)/combination ICI, anti-PD1 monotherapy	38	% relapse, time to PD, OS	8% relapse rate after ICI cessation and similar RRs and time to PD in patients who discontinue electively compared to TLTs.	MA	8
16	Dutheil et al., 2021 (36)	France (Single center, Goustave Roussy, 2010 – 2020)	Retrospective observational study	141/133 ^^^ (CR)	Metastatic setting (NA)/type of ICI not defined	42	% relapse, rechallenge	13% relapse rate post discontinuation for CR and 46% DCR after ICI rechallenge. Mucosal or acral, wild type tumors and patients who received prior treatments demonstrated increased risk for relapse.	MA	6
17	Gibney et al., 2021 (35)	USA (Single center, Georgetown, 2013- 2019)	Retrospective observational study	122/52 (disease control with and without toxicity)	Metastatic setting (prior lines)/combination ICI, anti-PD1 monotherapy	34	% relapse, time to PD, Rechallenge, OS	15.3% RRs after ICI cessation and higher rates in patients with TLTs. Mucosal melanomas showed shorter time to PD compared to other histologic subtypes.	MA	8
18	Pokorny et al., 2021 (37)	USA (Single center, 2015 – 2018)	Retrospective observational study	52/52 (disease control)	Metastatic setting (1^st^ line)/Anti-PD1 monotherapy	11.1 (95%CI 10.5 – 11.4)	% relapse, time to PD, rechallenge, PFS	25% relapse rate within 3.9 mo and 100% DCR after rechallenge. Younger age, history of brain metastasis and post- PD1 LDH were significant predictors of recurrence.	MA	9
19	Schank et al., 2021 (51)$	Germany (3 centers,	Retrospective observational study	45/45 (disease control or TLTs)	Metastatic setting (prior lines)/combination ICI, anti-PD1 monotherapy	34	% relapse	20% relapse rate after cessation and patients with CMR demonstrated prolonged time to PD compared to non-CMR patients.	MA	7
20	Van Zeijl et al., 2021 (11)	The Netherlands (DMTR registry, 2014 – 2017)	Retrospective observational study	324 (CR,PR,SD patients)/324 (CR, TLT, patient choice)	Metastatic setting (1st line)/anti-PD1 monotherapy	NA	% relapse, rechallenge, time to PD, PFS, OS	26% relapse rate within median 9.5 mo post cessation in patients with CR. BOR and reason for discontinuation were significant predictors of PFS and OS.	MA	8
21	Valentin et al., 2021 (38)	France (Single center, 2014 – 2019)	Retrospective observational study	604/65 (CR, elective, TLTs)	Metastatic setting (prior lines)/anti-PD1 monotherapy	36.5	% relapse, rechallenge, time to PD,	18% relapse rate with similar percentages in patients who discontinued electively or due to TLTs. 40% DCR rate after rechallenge. No significant predictors of relapse post-discontinuation were detected.	MA	8
22	Betof-Warner et al., 2020 (39)	USA (Single center, MSKCC 2009 – 2018)	Retrospective observational study	396/97 (CR)	Metastatic setting (prior lines)/Anti-PD1 monotherapy	28.9	% relapse, rechallenge, PFS, OS	23.7% relapse rate and 20% DCR rate after rechallenge. Time to CR was not associated significanlty with relapse.	MA	7
23	Makela et al., 2020 (50)	Finland (Single center, Helskinsi, 2015 – 2017)	Retrospective observational study	38/21 (patients with disease control who discontinued treatment)	Metastatic setting (prior lines)/Anti-PD1 monotherapy (nivolumab or pembrolizumab)	25	% relapse, Rechallenge,	71% relapse rate and 50% DCR after rechallenge, but with a median 3 months of treatment.	MA	5
24	Mesnard et al., 2020 (40)≠	France (Single center, Nantes, 2014 - 2018)	Retrospective observational study	87/26 (CR)	Metastatic setting (prior lines)/Anti-PD1 monotherapy (nivolumab)	31	% relapse, time to PD, rechallenge, PFS	29.1% relapse rate in patients with CR. Residual disease in PET/CT was significantly associated with relapse.	MA	7
25	Swami et al., 2020 (43)	USA (Single center, Iowa Hospital, 2012 – 2017)	Retrospective observational study	169/15 (TLTs)	Metastatic setting (prior lines)/Anti-PD1 monotherapy (nivolumab or pembrolizumab)	30.3	% relapse, time to PD, PFS, OS	53.3% relapse rates in patients with TLTs. Median OS not reached due to durable clinical benefit.	MA	7
26	Tikkanen et al., 2020 (52)	Finland (Single center, 2014 – 2019)	Retrospective observational study	30/14 (disease control)	Metastatic setting (prior lines)/Anti-PD1 monotherapy	5	Time to PD, rechallenge, OS	Relapse within median 23 months after discontinuation and 25% DCR after rechallenge.	MA	6
27	Warburton et al., 2020 (41)	Australia (Single center, Perth, 2013 – 2019)	Retrospective observational study	70/70 (Disease control)	Metastatic setting (prior lines)/Anti-PD1 monotherapy (Pembrolizumab)	11.8	% relapse, Time to PD after cessation, PFS, OS	18.5% relapse rate within median 11.1 months post-cessation.	MA	7
28	Bisschop et al.,2019 (23)	The Netherlands	Retrospective observational study	147/5 (TLT)	Metastatic setting (prior treatments)/Anti-PD1 monotherapy (Pembrolizumab)	37	24-month OS rate	Similar 24-month OS rates for patients who discontinued early due to AEs compared to patients who continued treatment	SR	6
29	Gauci et al., 2019 (49)@	France (Single center, Goustave Roussy, 2011 – 2017)	Retrospective analysis of patients in phase I trial	76/17 (disease control)	Metastatic setting (prior lines)/Anti-PD1 monotherapy (nivolumab or pembrolizumab)	34 *(total cohort)*	% relapse	17.6% relapse rate post-cessation in patients with disease control.	MA	7
30	Hamid et al., 2019 (KEYNOTE 001) (2)	Multicenter	Phase Ib, open- label clinical trial	655/72 (disease control, CR/PR)	Metastatic setting (prior lines)/Anti-PD1 monotherapy (pembrolizumab)	55	% relapse, time to PD, rechallenge	9.7% relapse rate after elective discontinuation. 11.1 months from cessation to relapse and 50% DCR after rechallenge.	MA	~
31	Handa et al., 2019 (46)	Single center (Japan, 2014 – 2018)	Retrospective observational study	4/4 (CR, separate for TLTs)	Metastatic setting (1^st^ line)/Anti-PD1 monotherapy (Pembrolizumab	NA	% relapse	None patient relapsed after discontinuation of ICI for CR.	MA	4
32	Jansen et al., 2019 (42)	Multicenter (Europe and Australia, 2013-2016)	Retrospective observational study	185/185 (disease control, absence of AEs)	Metastatic setting (prior lines)/Anti-PD1 monotherapy (nivolumab or pembrolizumab)	32	% relapse, time to PD, rechallenge, PFS	21.6% relapse rate and 12 months median time to relapse. CR at discontinuation was associated with decreased risk for relapse.	MA	9
33	Robert et al., 2019 (KEYNOTE 006) (6)	Multicenter	Open – label, randomized phase III clinical trial	556/103 (treatment study completion)	Metastatic setting (prior lines)/Anti-PD1 monotherapy (pembrolizumab)	57.7	% relapse, time to PD, rechallenge, PFS, OS	26.2% relapse rate within 33.3 months post-cessation. BOR during treatment and duration of treatment were significant predictors of PFS post discontinuation.	MA	~
34	Bernard-Tessier et al., 2018 (22)@	France (Single center, Goustave Roussy, 2011 – 2017)	Retrospective analysis of patients in phase I trial	1/1	Metastatic setting/anti-PD1 monotherapy	NA	Rechallenge	One patient who was on PR after discontinuation, rechallenged with anti-PD1 inhibitor and remained on PR.	SR	5
35	Saiag et al., 2018 (44)	France (Single center, Versailles, 2010 – 2020)	Retrospective observational study	134/19 (CR)	Metastatic setting (prior treatment)/Anti-PD1 monotherapy	NA	% relapse	None patient on CR relapsed after 13 months f.u. post discontinuation.	MA	7
36	Schvartsman et al., 2018 (47)	USA (MD Anderson, 2012 – 2016)	Retrospective observational study	580/75 (disease control or toxicity)	Metastatic setting (prior lines)/Anti-PD1 monotherapy (nivolumab or pembrolizumab)	NA	% relapse, Rechallenge	10.6% relapse rates and similar rates in patients who discontinued electively or due to TLTs.	MA	7
37	Ladwa et al., 2017 (45)	Australia (2 centers)	Retrospective observational study	29/29 (CR)	Metastatic setting (prior lines)/Anti-PD1 monotherapy (nivolumab or pembrolizumab)	10.4	% relapse, time to PD	11.1% RRs after cessation for CR.	MA	6
38	Topalian et al., 2014 (21)	Multicenter	Dose escalation, cohort expansion study of phase I trial	107/17 (disease control)	Metastatic setting (prior lines)/Anti-PD1 monotherapy (nivolumab)	NA	% relapse	71% of patients maintained their response after treatment cessation for disease control.	SR	5

Elective discontinuation include patients with CR, PR, SD and without toxicities.

~ Risk of bias assessment for RCTs is available at **Supplementary Material**, * same center, different time periods, overlapping populations cannot be excluded, ^ 94.3% interrupted treatment after CR, % Betof – Warner et al., TTF or OS were calculated from time of CR and not from time to treatment discontinuation, @ similar patient population, different outcomes, $ responses were evaluated using PET/CT, ≠Same population with Bocquet et al., 2019 (78).

RCT, randomized controlled trial; SR, systematic review; MA, meta-analysis; NOS, Newcastle – Ottawa scale; US, United States; TLTs, treatment-limiting toxicities; PD-1, programmed- cell death 1; PFS, progression-free survival; OS, overall survival; PD, progressive disease; NA, not available; DCR, disease control rate; CR, complete response; PR, partial response; SD, stable disease; ICI, immune checkpoint inhibitor; 95%CI, confidence interval; IQR, interquartile range; CMR, complete metabolic response

### 3.2 Relapse rates after ICI discontinuation

**Figure 2:**
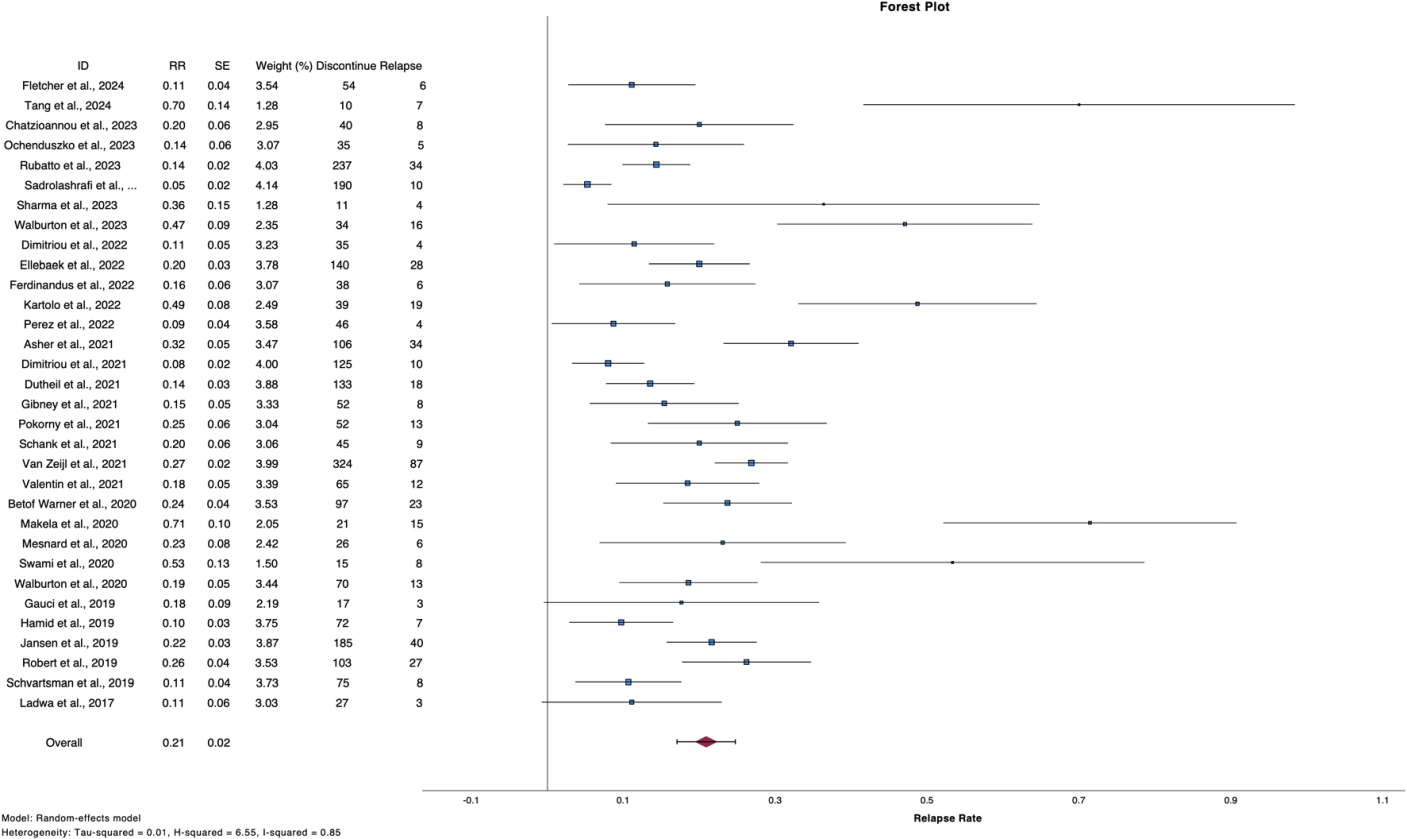
Forest plot demonstrate relapse rate after ICI discontinuation for reason other than PD.

2,542 patients discontinued treatment with ICIs electively or due to TLTs and from them, 495 patients experienced progression [number of studies (n)=34, RR 20.9%, 95%CI 17.1 – 24.7%, I^2^ 85%) (**Table 2**, **Figure 2**) (2, 6, 9–12, 24–51). Regarding reason for discontinuation, 28 studies reported data for elective cessation after disease control, leading to a pooled relapse rate of 15.9% (n=27, RR 15.8%, 95%CI 12.4-19.4%, I^2^ 72%), while for patients who discontinued after CR, relapse rate was smaller (n=22, RR 13.2%, 95%CI 10.5 – 16.0, I^2^ 43%). Nine studies provided data about patients who discontinue electively with BOR of PR/SD and concluded to a 27.7% relapse rate after ICI cessation (n=9, RR 27.7%, 95%CI 19.2 – 36.3, I^2^ 53%) (**Supplementary Table 4**, **Supplementary Figure 1**). Finally, patients with TLTs demonstrated higher rate of relapse post-cessation (n=14, RR 25.9%, 95%CI 18.3 – 33.4, I^2^ 70%) (**Supplementary Figure 2**).

**Table 2:** Studies referring to discontinuation of ICIs, reason for discontinuation, relapse rates and subgroup analysis based on reason for cessation.

Author, Year	Type of ICI use	Reason for discontinuation	Median treatment duration (mo)	Median f.u. post discontinuation (mo)	Relapse rate (n/N, %)	CR (n/N)	Electivediscontinuation (n/N)	TLTs (n/N)
Fletcher et al., 2024 (24)	Anti-PD1 monotherapy	Elective, TLTs, decline PS	NA	NA	6/54 (11.1)	–	–	–
Tang et al., 2024 (25)	Anti-PD1 monotherapy	Protocol completion	26.1 (range 22.2 – 29.9)	37.5 (range 35 – 39.3)	7/10 (70)	–	7/10	–
Chatzioannou et al., 2023 (26)	Combination ICI, anti-PD1 monotherapy	Complete response	22 (IQR 17-24)	47 (95%CI 38 – 51)	8/40 (20)	8/40	6/30	2/8
Ochenduszko et al., 2023 (10)	Anti-PD1 monotherapy	Complete response	23.4 (range 1.3 – 50.5)	24.1 (95%CI 17.9 – 30.5)	5/35 (14.3)	5/35	5/35	–
Rubatto et al., 2023 (9)	Anti-PD1 monotherapy	Complete response, TLTs, patient/physician choice	33 (range 1-98)	21 (range 1-81)	34/237 (14.3)	10/128	17/163	17/74
Sadrolashrafi et al., 2023 (28)	Combination ICI, anti-PD1 monotherapy	Complete response	8.91 (range 1.81 – 26.9)	60.2 (range 7.86 – 146)	10/190 (5.26)	10/190	10/190	–
Sharma et al., 2023 (29)	anti-PD1 monotherapy	Disease control (TLTs or not)	22 *	21.3*	4/11 (36.3)	–	2/6	2/5
Warburton et al., 2023 (48)	Combination ICI	TLTs	NA	NA	16/34 (47.1)	1/9	–	16/34
Dimitriou et al., 2022 (30)	Combination ICI, anti-PD1 monotherapy	TLTs, elective	NA	NA	4/35 (11.4)	–	1/9	3/26
Ellebaek et al., 2022 (31)	Combination ICI, anti-PD1 monotherapy	TLTs or elective discontinuation	7.8	29.3	28/140 (20)	–	17/92	11/48
Ferdinandus et al., 2022 (32)	Combination ICI, anti-PD1 monotherapy	TLTs or elective discontinuation	19 (0.7 – 48)	37.3	6/38 (15.8)	2/13	4/27	2/11
Kartolo et al., 2022 (27)	Combination ICI, anti-PD1 monotherapy	TLTs or elective discontinuation	NA	NA	19/39 (48.7)	–	14/27	5/12
Perez et al., 2022 (12)	Combination ICI, anti-PD1 monotherapy	CR	9.6	26	4/46 (8.7)	4/46	4/46	–
Asher et al., 2021 (34)	Combination ICI, anti-PD1 monotherapy	CR, elective, TLTs	15.2	20.8	34/106 (32.1)	19/80	–	26/60
Dimitriou et al., 2021 (33)	Combination ICI, anti-PD1 monotherapy	CR or TLTs with CR	16	NA	10/125 (8)	7/68^	7/68	3/39
Dutheil et al., 2021 (36)	type of ICI not defined	CR	NA	42	18/133 (13.5)	18/133	18/133	–
Gibney et al., 2021 (35)	Combination ICI, anti-PD1 monotherapy	Elective or TLTs	12.1/3.7	–	8/52 (15.4)	–	2/24	6/28
Pokorny et al., 2021 (37)	Anti-PD1 monotherapy	Elective without TLTs	11.1 (95%CI 10.5 – 11.4)	20.5	13/52 (25)	2/13	13/52	–
Schank et al., 2021 (51)	Combination ICI, anti-PD1 monotherapy	Elective or TLTs	21 (range, 1 – 42)	34	9/45 (20)	–	–	–
Van Zeijl et al., 2021 (11)	Anti-PD1 monotherapy	CR, elective discontinuation	11.8	NA	87/324 (26.8)	16/90	16/90	–
Valentin et al., 2021 (38)	Anti-PD1 monotherapy	CR, elective, TLT	14.1 (range, 0.7 – 51.2)	15.7 (range, 2.5 – 45.1)	12/65 (18.4)	3/25	5/37	7/28
Betof Warner et al., 2020 (39)	Anti-PD1 monotherapy	CR, TLTs	9.4	NA	23/97 (23.7)	–	–	–
Makela et al., 2020 (50)	Anti-PD1 monotherapy	Protocol completion	3 (range, 0 - 6)	NA	15/21 (71.4)	–	–	–
Mesnard et al., 2020 (40)	Anti-PD1 monotherapy	CR	8.5	NA	6/26 (23.1)	6/26	6/26	–
Swami et al., 2020 (43)	Anti-PD1 monotherapy	TLTs	4.7 (range, 0.7 – 11.5)	NA	8/15 (53.3)	1/8		8/15
Warburton et al., 2020 (41)	Anti-PD1 monotherapy	Disease control	11.8 (range, 3 – 33)	34.2 (2 – 70.8)	13/70 (18.6)	9/61	9/61	–
Gauci et al., 2019 (49)	Anti-PD1 monotherapy	Disease control	NA	NA	3/17 (17.6)	–	3/17	–
Hamid et al., 2019 (2)	Anti-PD1 monotherapy	Protocol completion	23.7 (range, 8.27 – 50.2)	NA	7/72 (9.7)	6/67	7/72	–
Handa et al., 2019 (46)	Anti-PD1 monotherapy	CR	NA	NA	0/4 (0)	0/2	0/2	0/2
Jansen et al., 2019 (42)	Anti-PD1 monotherapy	Disease control	12 (range, 0.7 – 43)	18 (0.7 – 48)	40/185 (21.6)	16/117	40/185	–
Robert et al., 2019 (6)	Anti-PD1 monotherapy	Protocol completion		34.2 (IQR 33.3 – 36.1)	27/103 (26.2)	5/21	27/103	–
Saiag et al., 2018 (44)	Anti-PD1 monotherapy	CR	14.5	13	0/19 (0)	0/19	0/19	–
Schvartsman et al., 2019 (47)	Anti-PD1 monotherapy	Elective or toxicity	Elective: 19.6 mo Toxicity: 6.5	16	8/75 (10.7)	–	3/41	5/34
Ladwa et al., 2017 (45)	Anti-PD1 monotherapy	CR	10.4mo	NA	3/27 (11.3)	3/27	3/27	–

* time for the total cohort.

N, number of patients who discontinued treatment with ICI; n, number of patients who relapsed after discontinuation; %, percentage of patients; mo, months; TLTs, treatment-limiting toxicities; PD-1, programmed- cell death; DCR, disease control rate; CR, complete response; ICI, immune checkpoint inhibitor; TLTs, treatment-limiting toxicities; PS, performance status; NA, not available; 95%CI, confidence interval.

### 3.3 Time to relapse

**Figure 3:**
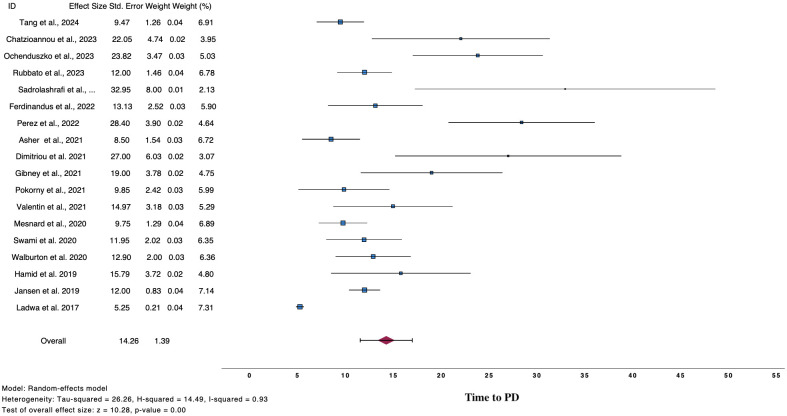
Forest plot demonstrate time to PD after ICI cessation for reasons other than PD.

Twenty-two studies analyzed time to PD post discontinuation (**Table 3**), 18 provided extractable data and the pooled mean time to PD was 14.26 months (n=18, mean time 14.26, 95%CI 11.54 – 16.98, I^2^ 93%) (**Figure 3**) (2, 9, 10, 12, 25, 26, 28, 32–35, 37, 38, 40–43, 45). Similar results were drawn, when the analysis was restricted to 140 patients, whose data were extracted from swimmer plots only (n=14, mean time 14.61 months, 95%CI 12.31 – 16.91) (2, 10, 12, 25, 26, 28, 32, 34, 35, 37, 38, 41, 42, 45). In addition, patients who ceased treatment for CR demonstrated numerically higher mean time to PD (n=13, mean time 15.88, 95%CI 12.29 – 19.47) compared to overall population and patients with TLTs (**Supplementary Figure 3**).

**Table 3:** Time to progressive disease post cessation in overall population and according to reason for discontinuation.

Author, Year	Number of patients who discontinue/who relapse	Type of ICI use	Median treatment duration (mo)	Median time to PD post cessation (mo)	CR (mo)	Elective discontinuation (mo)	TLTs (mo)
Tang et al., 2024 (25)	10/7	Anti-PD1 monotherapy	26.1 (range 22.2 – 29.9)	12.1 (range 1-12.7)	–	12.1 (range 1-12.7)	–
Chatzioannou et al., 2023 (26)	40/8	Combination ICI, anti-PD1 monotherapy (separate analysis)	22	15.3 (range 5.6 – 52)	15.3 (range 5.6 – 52)	18.3 (6.1 – 51.8)	–
Ochenduszko et al., 2023 (10)	35/5	Anti-PD1 monotherapy	23.4 (range 1.3 – 50.5)	25.9 (range 8.30 – 35.2)	25.9 (range 8.30 – 35.2)	25.9 (range 8.30 – 35.2)	–
Rubatto et al., 2023 (9)	237/34	Anti-PD1 monotherapy	33 (range 1 – 81)	12 (range 1-35)	–	–	–
Sadrolashrafi et al., 2023 (28)	190/10	Combination ICI, anti-PD1 monotherapy (separate analysis)	8.91 (range 1.81 – 26.9)	17.2 (range 4.9 – 92.5)	17.2 (range 4.9 – 92.5)	17.2 (range 4.9 – 92.5)	–
Ferdinandus et al., 2022 (32)	38/6	Combination ICI, anti-PD1 monotherapy (separate analysis)	19.7 (range 0.7 – 48)	12.8 (range 2.78 – 24.16)	13.4 (range 2.5 – 24)	15.1 (2.5 – 24.2)	10
Kartolo et al., 2022 (27)	39/19	Combination ICI, anti-PD1 monotherapy	NA	NA	–	19.7	25.1
Perez et al., 2022 (12)	46/4	Combination ICI, anti-PD1 monotherapy	9.6	27.4 (range 15.9 – 42.9)	27.4 (range 15.9 – 42.9)	27.4 (range 15.9 – 42.9)	–
Asher et al., 2021 (34)	106/34	Combination ICI, anti-PD1 monotherapy	15.2	8.5 (range, 1.5 – 37.5)	12 (range, 2.97 – 37.2)	–	–
Dimitriou et al., 2021 (33)	125/10	Combination ICI, anti-PD1 monotherapy (separate analysis)	16	21 (range, 0.1-66)	19 (range, 0.1-42)	19 (range, 0.1-42)	25 (range, 0.1-66)
Gibney et al., 2021 (35)	52/8	Combination ICI, anti-PD1 monotherapy	7.12	18.5 (range, 1-38)	–	29 (range, 20-38)	13.5 (range, 1 – 25)
Pokorny et al., 2021 (37)	52/13	Anti-PD1 monotherapy	11.1	3.9 (range, 0.7 – 30.9)	16.7 (range, 8.5 – 24.9)	3.9 (range, 0.7 – 30.9)	–
Van Zeijl et al., 2021 (11)	324/87	Anti-PD1 monotherapy	11.8	CR: 9.5 PR: 7.5 SD: 5.1:	9.5	–	–
Valentin et al., 2021 (38)	65/12	Anti-PD1 monotherapy	14.1	9 (range, 1.9 – 40.9)	9.3 (range, 4 – 11.9)	9.1 (range, 3.5 – 12.1)	7.1 (range, 1.9 – 40.9)
Mesnard et al., 2020 (40)	26/6	Anti-PD1 monotherapy	8.5	11 (range,3 – 14)	11 (range, 3 – 14)	11 (range, 3 – 14)	–
Swami et al., 2020 (43)	15/8	Anti-PD1 monotherapy	4.7	13.3 (range, 0.7 – 20.5)	15.6		13.3
Tikkanen et al., 2020 (52)	14/NA	Anti-PD1 monotherapy	NA	23 (95%CI 2.6 – 34)	–	–	–
Warburton et al., 2020 (41)	70/13	Anti-PD1 monotherapy	11.8	11.1 (range, 2.2 - 27.2)	13.72 (range, 3.46 – 22.42)	13.72 (range, 3.46 – 22.42)	–
Hamid et al., 2019 (2)	72/7	Anti-PD1 monotherapy	23.7	11.1 (range, 3.46 – 37.5)	12.8 (range, 5.46 – 37.5)	11.1 (range, 3.46 – 37.5)	–
Jansen et al., 2019 (42)	185/40	Anti-PD1 monotherapy	12	12 (range, 2 – 23)	12.1 (range, 1.97 – 23.1)	12 (range, 2 – 23)	–
Robert et al., 2019 (6)	103/27	Anti-PD1 monotherapy	NA	33.3 (IQR 26 – NR)	–	–	–
Ladwa et al., 2017 (45)	27/3	Anti-PD1 monotherapy	10.4	5.6 (range, 4.9 – 6)	5.6 (range, 4.9 – 6)	5.6 (range, 4.9 – 6.3)	–

mo, months; TLTs, treatment-limiting toxicities; PD-1, programmed- cell death; CR, complete response; ICI, immune checkpoint inhibitor; TLTs, treatment-limiting toxicities; PS, performance status; NA, not available; 95%CI, confidence interval; IQR, interquartile range.

Regarding factors affecting the time to PD after cessation, 18 studies investigated the role of clinical characteristics of patients and the tumor (i.e. age, gender, anatomic location, histologic subtype), type of ICI used and response to treatment on relapse, and were synthesized qualitatively (6, 9–11, 26, 27, 32, 33, 35–42, 47, 51). Patients with mucosal melanoma had shorter time to PD compared to patients with other histologic subtypes, while CR at discontinuation was associated with decreased risk and prolonged time to PD compared to TLTs (**Supplementary Table 5**).

### 3.4 Rechallenge after relapse

**Figure 4:**
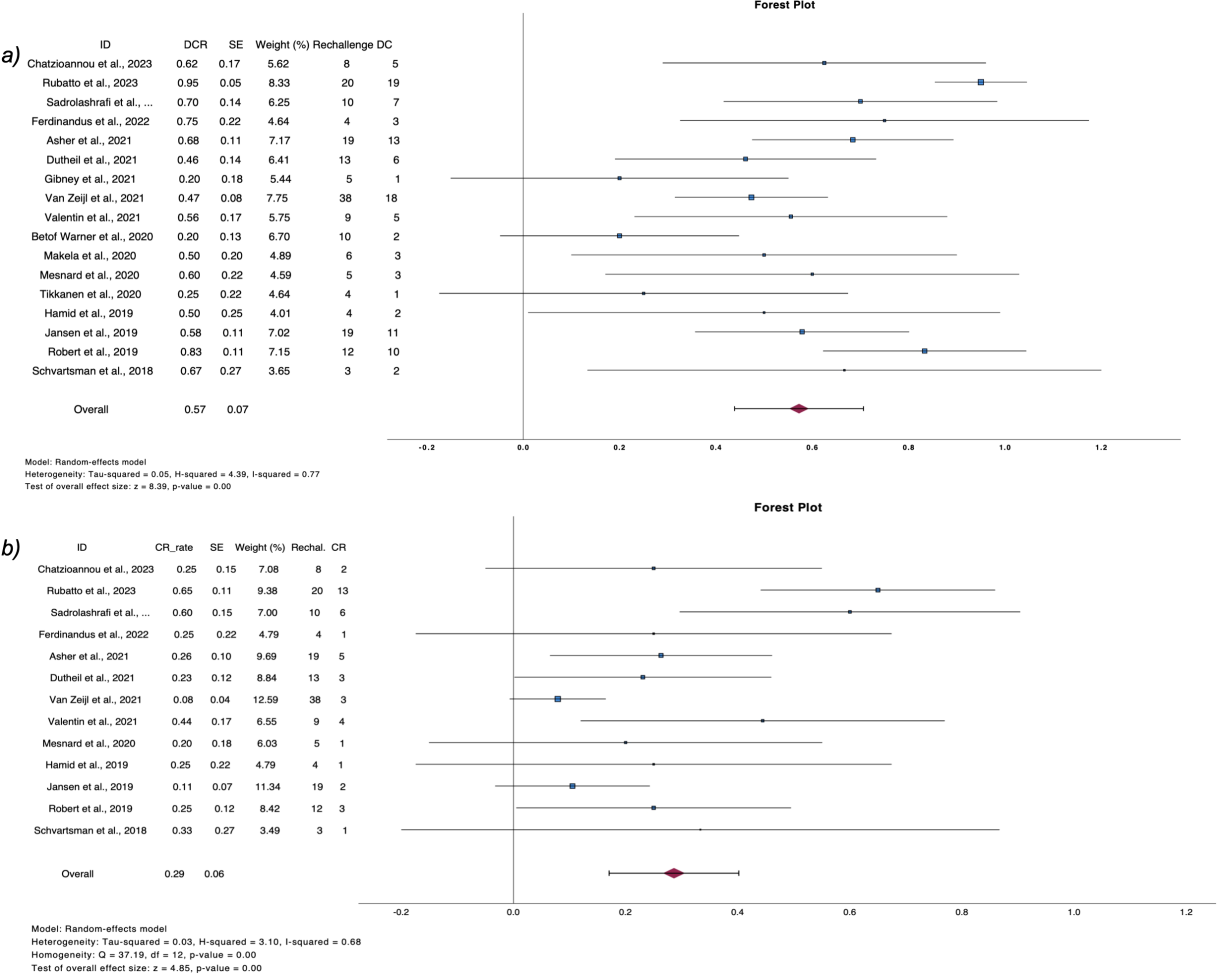
Forest plots demonstrate **(A)** disease control rate and **(B)** complete response rate after ICI rechallenge following PD.

Post relapse management of patients who progressed after discontinuation is summarized in **Table 4**. From 380 patients with PD, 208 received a second course of ICIs, including both anti-CTL4 and anti-PD1 or anti-PD1 monotherapy (2, 6, 9, 11, 25, 26, 28, 29, 32–40, 42, 45, 47, 50, 52). Pooled CR rate after rechallenge was 28.6% (n=16, CR rate 28.6%, 95%CI 17.1 – 40.2, I^2^ 68%), while 57.3% of patients exhibited disease control (n=22, DCR rate 57.3%, 95%CI 43.9 – 70.6, I^2^ 73%) (**Figure 4**).

**Table 4:** Rechallenge with ICIs after progression, disease control rate and BOR after rechallenge.

Author, Year	Type of ICI use	Reason for discontinuation	Patients who ceased treatment/patients relapse	Rechallenge	Median time to rechallenge after relapse post discontinuation *	Disease control rate (DCR) after rechallenge (n,%)	BOR after rechallenge
Tang et al., 2024 (25)	Anti-PD1 monotherapy	Protocol completion	10/7	4 (Anti-PD1 monotherapy)	Rechallenge of ICI started immediately after disease progression post cessation in all 4 patients.	4 (100)	4 PR
Chatzioannou et al., 2023 (26)	Combination ICI, anti-PD1 monotherapy	Complete response	40/8	8 (anti-PD1 monotherapy or anti-CTL4 or combination ICI)	Rechallenge of ICI started immediately after disease progression post cessation in 7/8 patients. One patient received 2^nd^ course after 6 months post relapse.	5 (62.5)	2 CR, 3 PR, 3 PD
Rubatto et al., 2023 (9)	Anti-PD1 monotherapy	Complete response, TLTs, patient/physician choice	237/34	20 (anti-PD1 monotherapy)	NA	19 (95)	13 CR, 4 PR, 2 SD, 1 PD
Sadrolashrafi et al., 2023 (28)	Combination ICI, anti-PD1 monotherapy	Complete response	190/10	5 (ICI only)	NA	4 (80)	4 CR, 1 PD
Sharma et al., 2023 (29)	anti-PD1 monotherapy	Disease control (TLTs or not)	11/4	2 (anti-PD1 monotherapy)	NA	0	2 PD
Ferdinandus et al., 2022 (32)	Combination ICI, anti-PD1 monotherapy	TLTs or elective discontinuation	38/6	4 (Combination ICI, anti-PD1 monotherapy)	Rechallenge of ICI started immediately after disease progression post cessation in 3/4 patients. One patient received 2^nd^ course after 3 months post relapse.	3 (75)	1 CR, 1 PR, 1 SD, 1 PD
Asher et al., 2021 (34)	Combination ICI, anti-PD1 monotherapy	CR, elective, TLTs	106/34	19 (anti-PD1 monotherapy)	NA	13 (68.4)	5 CR, 4 PR, 4 SD, 4 PD
Dimitriou et al., 2021 (33)	Combination ICI, anti-PD1 monotherapy	CR or TLTs with CR	125/10	5 (Combination ICI, anti-PD1 monotherapy)	NA	5 (100)	NA
Dutheil et al., 2021 (36)	type of ICI not defined	CR	133/18	13 (ICI)	NA	6 (46.1)	3 CR, 2 PR, 1 SD
Gibney et al., 2021 (35)	Combination ICI, anti-PD1 monotherapy	Elective or TLTs	52/8	5 (in combination with other modalities)	NA	1 (20)	NA
Pokorny et al., 2021 (37)	Anti-PD1 monotherapy	Elective without TLTs	52/13	7 (anti-PD1 monotherapy)	NA	7 (100)	NA
Van Zeijl et al., 2021 (11)	Anti-PD1 monotherapy	CR, elective discontinuation	324/87	38 (combination ICI, anti-PD1 monotherapy)	NA	18 (47.4)	3 CR, 8 PR, 7, SD, 12 PD
Valentin et al., 2021 (38)	Anti-PD1 monotherapy	CR, elective, TLT	65/12	9 (anti-PD1 monotherapy)	NA	5 (55.6)	4 CR, 1 SD, 4 PD
Betof Warner et al., 2020 (39)	Anti-PD1 monotherapy	CR, TLTs	97/23	10 (combination ICI, anti-PD1 monotherapy)	NA	2 (20)	NA
Makela et al., 2020 (50)	Anti-PD1 monotherapy	Protocol completion	21/15	6 (anti-PD1 monotherapy)	NA	3 (50)	3 RR
Mesnard et al., 2020 (40)	Anti-PD1 monotherapy	CR	26/6	5 (anti-PD1 monotherapy)	NA	3 (60)	1 CR, 2 SD, 2 PD
Tikkanen et al., 2020 (52)	Anti-PD1 monotherapy	Disease control	14/NA	4 (anti-PD1 monotherapy)	NA	1 (25)	1 SD, 3 PD
Hamid et al., 2019 (2)	Anti-PD1 monotherapy	Protocol completion	72/7	4 (anti-PD1 monotherapy)	Rechallenge of ICI started after median 1.2 months (range 0.97 – 1.33) post progression.	2 (50)	1 CR. 1 SD. 2 PD
Jansen et al., 2019 (42)	Anti-PD1 monotherapy	Disease control	185/40	19 (anti-PD1 monotherapy)	Rechallenge of ICI started immediately after disease progression post cessation in all 19 patients.	11 (57.9)	2 CR, 4 PR, 5 SD, 6 PD
Robert et al., 2019 (6)	Anti-PD1 monotherapy	Protocol completion	103/27	12 (anti-PD1 monotherapy)	Rechallenge of ICI started immediately after disease progression post cessation in 4/11 patients. 7 patients received 2^nd^ course after median 3 months (range 1.62 – 8.98 months)	10 (83.3)	3 CR, 4 PR, 3 SD
Schvartsman et al., 2018 (47)	Anti-PD1 monotherapy	Elective, TLTs	75/8	3 (anti-PD1, anti-CTL4)	NA	2 (66.7)	1 CR, 1 PR, 1 PD
Ladwa et al., 2017 (45)	Anti-PD1 monotherapy	CR	27/3	1 (anti-PD1)	NA	1 (100)	NA

mo, months; TLTs, treatment-limiting toxicities; PD-1, programmed- cell death; CR, complete response; ICI, immune checkpoint inhibitor; TLTs, treatment-limiting toxicities; PS, performance status; NA, not available; 95%CI, confidence interval; IQR, interquartile range; PR, partial response; SD, stable disease.

*Data available from swimmer plots of original studies.

### 3.5 PFS – OS

PFS and OS for patients who ceased treatment were analyzed qualitatively, along with factors affecting time to PD and the results were presented in **Supplementary Table 6**. Elective treatment discontinuation or due to CR, led to higher OS rates compared to treatment cessation due to TLTs.

### 3.6 Subgroup analysis

#### 3.6.1Type of ICI used

Patients were treated either with combination ICI or anti-PD1 monotherapy in 12 studies and from them, 7 provided extractable data (12, 26, 32–35, 48), concluding to 18.7% pooled RR (n=7, RR 18.7%, 95%CI 7.4 – 30.3). Contrary to, 1,792 patients were treated with anti-PD1 monotherapy, and 20.8% relapsed post-cessation (n=7, RR 20.8%, 95%CI 16.5 – 24.9). Risk of relapse did not reach statistical significance when anti-CTL4+anti-PD1 treatment was compared to anti-PD1 monotherapy (n=7, OR 1.29, 95%CI 0.69 – 2.41).

#### 3.6.2 Type of studies

Higher pooled RR was detected in RCTs (n=3, RR 30.0%, 95%CI 9.0 – 50.4%) (2, 6, 25) compared to observational studies (n=31, RR 20.4%, 95%CI 16.5 – 24.4%) in the overall population. On the other hand, similar RRs were found between type of study when the analysis was restricted to patients with CR, or who had an elective discontinuation.

CR rates after rechallenge with ICI did not differ among various study types, while a second course of ICI led to higher DCR in RCTs (n=3, DCR 70.4%, 95%CI 45.3 – 98.6%) (2, 6, 25) compared to observational studies.

### 3.7 Sensitivity analysis

Through leave one-out sensitivity analysis and exclusion of low-quality studies (as assessed by NOS scale and GRADE checklist), the primary and secondary outcomes remained unaltered.

### 3.8 Meta-regression analysis

Duration of treatment before cessation was investigated as a predictor of relapse rate or time to PD in meta-regression analysis, concluding to a non-statistically significant association in both outcomes (**Supplementary Figures 4**-**6**).

### 3.9 Publication bias

Funnel plots regarding relapse rate and time to PD did not demonstrate a uniform distribution, implying possible small studies effect. This was not the case for DCR (Egger’s test, p>0.20) (**Supplementary Figure 7**).

## 4 Discussion

Survival outcomes after ICI treatment cessation for reasons other than disease progression or relapse in patients with advanced melanoma remains an unanswered question in medical literature (53–55). Our analysis shows a 20.9% relapse rate after a mean of 14.26 months post cessation. In addition, lower rates were found in patients who stopped treatment electively compared to patients who stopped therapy due to TLTs. When patients had PD after therapy cessation, reintroducing ICI led to a substantial DCR, including CRs in approximately one third of cases.

The optimal duration of treatment with ICI remains a matter of great debate in the literature (5, 56). As far as we know, there is no clinical, biological or scientific rationale to continue therapy until PD or to stop after 2 years. Still, although RCTs employed different schedules for total treatment duration, a substantial number of patients who ceased treatment for reasons other than PD will not recur during follow-up (1, 2, 6, 57). According to our analysis, approximately four out of 5 patients who discontinue treatment will not recur and this rate is line with relapse rates reported in observational studies and RCTs. Patients included in KEYNOTE-001 and KEYNOTE-006 trials, ceased treatment for CR or after completing 2 years of pembrolizumab, resulting in a 9.2% and 26.2% relapse rates respectively (2, 4, 6). Similar results were drawn from multicenter retrospective registry-based studies (9, 11, 42). Those findings hold significant implications, especially in CR patients, as treatment cessation could be associated with lower rates of chronic or delayed irAEs and reduced financial toxicity from prolonged ICI treatment.

Several factors influencing relapse rates after therapy discontinuation have been studied, particularly in the lack of reliable predictive biomarkers for safe ICI cessation. Dose-response and exposure-response curves of anti-CTL4 anti-PD1 inhibitors provided useful insights regarding the effect of treatment duration on outcomes. Recently published long-term survival data from randomized phase III trials, highlighted that patient who completed ≥94 weeks of treatment in KEYNOTE-006 or remained progression free at 3 years in CheckMate-067, maintained durable clinical benefit from ICI treatment (3, 4). In concordance, pharmacokinetic studies of nivolumab have demonstrated that receptor occupancy can be achieved with low doses, which persist for a significantly longer duration than the antibody’s half-life at given (58, 59). This suggest that efficacy is maintained independently of dose and treatment duration, which was verified also by our results, where duration of ICI treatment was not a significant predictor of relapse or time to PD post cessation in meta-regression analysis. However, real world data of patients with CR or PR from the EUMelaReg registry proved that prolonged ICI treatment led to longer PFS, compared to early cessation (<6 months), and patients with PR were mainly benefited (60). On the contrary, Lodde et al., reported no difference in outcomes of patients who achieved early responses to ICI compared to late, and these were not associated with the duration on treatment (61).

Those findings underscore the importance of the depth of response to treatment rather than the duration of treatment, as a biomarker of long-term preservation of response post-cessation. As demonstrated in the final analysis of CheckMate 067, patients who achieved at least 80% reduction in tumor size experienced long-term survival regardless of treatment with anti-CTL4 and anti-PD1 or anti-PD1 monotherapy (3). Similar findings were evident from our meta-analysis, where patients with CR demonstrated the lowest relapse rates, while type of treatment used did not influence significantly the risk of relapse, in line with findings from Pala et al. (13) and observational studies (26). In addition, retrospective multicenter observational studies concluded to a lower risk of relapse in patients who achieved CR on primary treatment compared to patients who ceased therapy with BOR of PR/SD or due to TLTs (9, 10, 42). The above are in line with our results, and highlights, especially for patients on PR/SD, the importance of careful co-estimation of disease-related factors along with the response to treatment, when ICI cessation is considered.

In contrast to better survival outcomes in patients who ceased ICI due to CR, our analysis found a survival disadvantage in patients with TLTs, characterized by higher relapse rates and shorter time to PD. Results about the effect of irAEs on outcomes of melanoma patients remain contradictory (62–65). While observational studies reported a prolonged PFS in patients who developed irAEs, implicating the presence of a strong immunogenic reaction against the tumor, our meta-analysis, along with other studies, did not verify that benefit after discontinuation (34, 62, 65). From a pathophysiologic perspective, the durability of ICI response is based on the generation of tissue-resident memory (TRM) CD8-T- cells (13). However, the possible use of high dose of corticosteroids and immunosuppressants for the management of high grade irAEs, especially in the early phase of ICI administration (66), an cause inhibitory effect on TCR affinity and decrease the production of effective memory cells, which may justify the increased relapse rate post-cessation (67–69).

In our analysis the mean time to PD after ICI cessation was estimated to be 14.26 months, with studies in the literature reporting similar times from 3.9 (37) to 33.3 months (6). The estimation of that time is considered clinically relevant for two reasons. First, a numerically longer time to PD in patients who ceased for CR compared to TLTs, possibly corroborate the aforementioned pathophysiologic mechanisms associated with relapse post cessation. In concordance, disease-related factors, such as histologic subtype, could influence the time to PD, as shown by our quantitative synthesis, where mucosal melanomas demonstrated shorter time to PD compared to other histologies. This could be explained by the distinct biologic characteristics between subtypes, with mucosal lesions exhibiting more aggressive biologic course, absence of UV signature and lower mutational burden, which could explain the lower efficacy of ICIs in this subtype and the shorter time to PD compared to cutaneous lesions (70, 71). Moreover, accurate estimation of the interval between ICI cessation and PD is crucial for implementing effective surveillance strategies in these patients, especially considering the importance of early detection of recurrence in patients where consensus on the follow-up scheme has not been reached. Simultaneously, the emergence of imaging- (i.e. FDG PET/CT) and blood-based biomarkers (i.e. ctDNA), combined with the knowledge of time to relapse, could provide valuable tools not only for patient monitoring, but also for decision making regarding treatment continuation or intensification in high-risk patients (31, 32, 48).

Following progression after cessation, a second course of ICI could be considered a reasonable option, especially in the context of limited therapeutic choices in patients with BRAF-wild type melanomas. Our analysis showed a 57.9% DCR and 28.9% after ICI (of any type) rechallenge, while in the literature, re-introducing ICI after PD resulted in a various ORRs, ranged from 20% (39) to 40% (72) and more than 60% (73, 74) and influenced by factors, such as the time of rechallenge after PD or the concomitant use of other treatments, such as radiotherapy. Our analysis included only patients who ceased treatment without progression, which means that, by definition, patients with primary resistance to treatment were excluded. Gang et al. summarized the evidence regarding rechallenge strategies in patients with NSCLC, pinpointing differences in pathophysiologic mechanisms according to reason for discontinuation (75). Consistent results were evident for melanoma also, reinforcing the concept of T-cell revitalization after rechallenge. This could potentially improve the ability of immune cells to recognize and eliminate tumor cells that escape from memory-T-cells surveillance (76). Although results from our analyses are promising, supporting rechallenge with ICI after PD, more sophisticated analyses on factors affecting ORRs, such as reason for cessation (including further analyses for patients who discontinue treatment on CR or for TLTs), type and sequence of ICI in rechallenge and BOR at first course are important, but were out of the scope of our analysis.

Our meta-analysis has several strengths. We reported the pooled RRs after ICI cessation, DCR after ICI rechallenge and the estimation of time to PD post discontinuation, which have not been reported before. Some limitations need to be mentioned. There was a high heterogeneity among studies both in primary and secondary outcomes, which is mainly attributed to different definitions of reason for discontinuation, the type of ICI investigated in each study and the different treatment duration. To minimize this heterogeneity, we conducted several subgroup and sensitivity analyses. However, we didn’t see significant changes in our primary results. Another limitation of our study was that uveal melanoma was an exclusion criterion. There are distinct biologic characteristics between histologic subtypes and, because of that, most of clinical trials and observational studies published in the literature included only patients with cutaneous melanoma, with uveal melanoma being actively excluded, especially in clinical trials. For those reasons, we decided to focus on cutaneous melanoma only, aiming to synthesize the evidence for the most reported subtype and simultaneously aiming to avoid higher heterogeneity in our results.

## 5 Conclusions

Discontinuation of immunotherapy for reasons other than PD could be a reasonable option for patients diagnosed with stage IV melanoma, possibly mitigating the risk of chronic irAEs and the economic burden of prolonged ICI therapy. Time and risk of PD after therapy discontinuation seem to be affected mostly by disease-related factors. A second course of ICI at time of PD remains considerable. Future clinical trials supported by more real-world evidence may help to answer the question about the optimal duration of ICI treatment in this setting (77).

## Data Availability

The original contributions presented in the study are included in the article/**Supplementary Material**. Further inquiries can be directed to the corresponding author.
