# Supplementary material for: Discontinuation of immune checkpoint inhibitors for reasons other than disease progression and the impact on relapse and survival of advanced melanoma patients. A systematic review and meta-analysis
Source: Front Immunol. 2025 Jan 31;16:1524945. doi: 10.3389/fimmu.2025.1524945 (PMC11825796; doi:10.3389/fimmu.2025.1524945)
Supplement: Supplementary file 1 [file DataSheet1.pdf]

Discontinuation of immune checkpoint inhibitors for reasons other than disease progression and the impact on relapse and survival of advanced melanoma patients. A systematic review and meta-analysis

Supplementary File

Search strategies used for literature search:

PubMed

("immunotherapy"[MeSH Terms] OR "immunotherapy"[Title/Abstract] OR "immune checkpoint inhibit*"[Title/Abstract] OR "ICI"[Title/Abstract] OR "ICB"[Title/Abstract] OR "anti-PD1"[Title/Abstract] OR "anti-PDL1"[Title/Abstract] OR "anti-CTL4"[Title/Abstract]) AND ("discontinu*"[Title/Abstract] OR "disconti*"[Text Word] OR "cessa*"[Title/Abstract] OR "stop*"[Title/Abstract] OR "premature disconti*"[Title/Abstract] OR "early disconti*"[Title/Abstract] OR "interrupt*"[Title/Abstract] OR "break"[Title/Abstract]) AND ("melanoma"[MeSH Terms] OR "melanoma"[Title/Abstract] OR "skin cancer"[Title/Abstract] OR "cutaneous melanoma"[Title/Abstract] OR ("melanoma"[MeSH Terms] OR "melanoma"[All Fields] OR "melanomas"[All Fields] OR "melanoma s"[All Fields]))

Scopus

TITLE-ABS-KEY (melanoma OR skin AND cancer OR "cutaneous melanoma") AND TITLE-ABS-KEY (immunotherapy OR "immune checkpoint inhibit*" OR ici OR icb OR anti-pd1 OR anti-pdl1 OR anti-ctl4) AND TITLE-ABS-KEY (disconti* OR cessation OR stop* OR interrupt* OR break)

Cochrane (CENTRAL)

ID	Search	Hits
#1	MeSH descriptor: [Melanoma] 3 tree(s) exploded	2734
#2	(melanoma):ti,ab,kw	6752
#3	(skin cancer):ti,ab,kw	9415
#4	MeSH descriptor: [Immunotherapy] explode all trees	12102
#5	immunotherapy	14670
#6	immune checkpoint inhibitor*	2233
#7	ICB	242
#8	ICI	1136
#9	(anti-PD1):ti,ab,kw	341
#10	(anti-PDL1):ti,ab,kw	90

#11 (anti-CTL4):ti,ab,kw 2

#12 disconti* 51175

#13 cessation 22187

#14 stop* 34715

#15 #5 OR #6 OR #7 OR #8 OR #9 OR #10 OR #11 17133

#16 #12 OR #13 OR #14 99704

#17 #1 OR #2 OR #3 14353

#18 #15 AND #16 AND #17 250

**Supplementary table 1:** Newcastle – Ottawa Scale for Risk of Bias assessment										
**Study**	**Year**	**Selection**				**Comparability**	**Outcome**			**Total**
		1	2	3	4		1	2	3	
Fletcher et al.	2024	b	a	a	a	A,b	b	a	d	8
Chatzioannou et al.	2023	b	a	d	a	A,b	d	a	b	7
Oczenduszko et al.,	2023	b	a	a	a	A,b	b	a	a	9
Rubatto et al.,	2023	b	a	d	a	A,b	b	a	d	7
Sadrolashrafi et al.,	2023	b	a	b	a	a	b	a	d	7
Sharma et al.	2023	b	a	d	a	a	b	a	d	6
Warburton et al.,	2023	b	a	b	a	a	b	a	d	6
Dimitriou et al.,	2022	b	a	d	a	A,b	b	a	d	7
Ellebaek et al.	2022	b	a	a	a	A,b	b	a	d	8
Ferdinandus et al.,	2022	b	a	a	a	A,b	b	a	d	8
Kartolo et al.,	2022	b	a	d	a	a	d	a	d	6
Perez et al.,	2022	b	a	a	a	a	b	a	d	7
Asher et al.,	2021	b	a	a	a	A,b	b	a	d	8
Dimitriou et al.,	2021	b	a	a	a	A,b	b	a	d	8
Dutheil et al.,	2021	b	a	d	a	A,b	d	a	d	6
Gibney et al.,	2021	b	a	a	a	A,b	b	a	d	8
Pokorny et al.,	2021	b	a	a	a	A,b	b	a	b	9
Schank et al.,	2021	b	a	a	a	a	b	a	d	7
Van Zeijl et al.,	2021	b	a	a	a	A,b	b	a	d	8
Valentin et al.,	2021	b	a	a	a	A,b	b	a	d	8
Betof – Warner et al.,	2020	b	a	d	a	A,b	b	a	d	7
Makela et al.	2020	b	a	d	b	a	b	a	d	5
Mesnard et al.,	2020	b	a	d	a	A,b	b	a	d	7
Swami et al.,	2020	b	a	a	a	a	b	a	d	7
Tikkanen et al.,	2020	b	a	d	a	a	b	a	d	6
Warburton et al.,	2020	b	a	a	a	a	b	a	d	7
Bisschop et al.,	2019	b	a	a	a	a	b	b	d	6
Gauci et al.,	2019	b	a	a	a	a	b	a	d	7
Handa et al.,	2019	c	a	d	a	a	d	a	d	4
Jansen et al.,	2019	b	a	a	a	A,b	b	a	b	9
Bernard-Tessier et al.,	2018	b	a	d	a	a	b	b	d	5
Saiag et al.	2018	b	a	a	a	a	b	a	d	7
Schvartsman et al.,	2018	b	a	a	a	a	b	a	d	7
Ladwa et al.,	2017	B	a	d	a	a	b	a	d	6
Topalian et al.,	2014	b	a	d	a	a	b	d	d	5

**Supplementary Table 2: Risk of bias assessment of Randomized controlled trials (RCTs) included in the study**							
	**Random sequence generation (selection bias**	**Allocation concealment (selection bias)**	**Blinding of participants and personnel (performance bias)**	**Blinding of outcome assessment (detection bias)**	**Incomplete outcome of data (attrition bias)**	**Selective reporting (reporting bias)**	**Other bias**
Tang et al., 2024	High	Low	High	High	Low	Low	Low
Hamid et al., 2019	Low	Low	High	Low	Low	Low	Low
Robert et al., 2019	Low	Low	High	Low	Low	Low	Low

Excluded studies from full – text

Reasons for exclusion

1. **Study population not according to inclusion criteria**
2. **Study design not according to inclusion criteria (including those without comparator group)**
3. **Outcomes of the study not according to inclusion criteria**

**Supplementary Table 3: Studies excluded from full-text screening with reasons**			
**No**	**Author**	**Year**	**Reason for exclusion**
1	DiGiacomo et al.	2024	Patient population and outcomes of the study not according to inclusion criteria
2	Dima et al.,	2024	Patient population not according to inclusion criteria
3	Janssen et al.	2024	Study design not according to inclusion criteria
4	Pala et al.,	2024	Study design not according to inclusion criteria
5	Stager et al.	2024	Patient population not according to inclusion criteria
6	Virtanen et al.,	2024	Patient population and outcomes of the study not according to inclusion criteria
7	Marron et al.	2023	Study design not according to inclusion criteria
8	Lodde et al.	2023	Patient population and outcomes of the study not according to inclusion criteria
9	Holmstroem et al.	2023	Patient population not according to inclusion criteria
10	Nardin et al.	2023	Patient population not according to inclusion criteria
11	Tachiki et al.	2023	Intervention of the study not according to inclusion criteria
12	Goodman et al.	2023	Outcomes of the study not according to inclusion criteria
13	Gupta et al.	2023	Outcomes of the study not according to inclusion criteria
14	Boutros et al.	2022	Study design not according to inclusion criteria
15	Cartun et al.	2022	Outcomes of the study not according to inclusion criteria
16	De Risi et al.	2022	Study design not according to inclusion criteria
17	Jansen et al.	2022	Study type not according to inclusion criteria
18	Kamminga et al.	2022	Outcomes of the study not according to inclusion criteria
19	Mantia et al.	2022	Outcomes of the study not according to inclusion criteria
20	Plazy et al.	2022	Study type not according to inclusion criteria
21	Schulz et al.	2022	Outcomes of the study not according to inclusion criteria
22	Villa-Crespo et al.	2022	Outcomes of the study not according to inclusion criteria
23	Wolchock et al.	2022	Outcomes of the study not according to inclusion criteria
24	De Meza et al.	2021	Patient population and outcomes of the study not according to inclusion criteria
25	Hodi et al.	2021	Outcomes of the study not according to inclusion criteria
26	Ksienski et al.	2021	Intervention of the study and outcomes not according to inclusion criteria
27	Manzano et al.	2021	Non-English paper
28	Mandala et al.	2021	Outcomes of the study not according to inclusion criteria
29	Musicco et al.	2021	Non-English paper
30	Regan et al.	2021	Outcomes of the study not according to inclusion criteria
31	Allouchery et al.	2020	Patient population and outcomes of the study not according to inclusion criteria
32	Banks et al.	2020	Study type not according to inclusion criteria
33	Carlino et al.	2020	Outcomes of the study not according to inclusion criteria
34	Machado et al.	2020	Outcomes of the study not according to inclusion criteria
35	Michielin et al.	2020	Study design not according to inclusion criteria
36	Horisberger et al.	2020	Study design not according to inclusion criteria
37	Robert et al.	2020	Study design not according to inclusion criteria
38	Sakakida et al.	2020	Outcomes of the study not according to inclusion criteria
39	Yang et al.	2020	Non-English paper
40	Parakh et al.	2019	Outcomes of the study not according to inclusion criteria
41	Regan et al.	2019	Patient population not according to inclusion criteria (Overlapping with other study)
42	Carlino et al.	2017	Study design not according to inclusion criteria

43	Horiguchi et al.	2017	Study design not according to inclusion criteria
44	Gandaghar et al.	2017	Outcomes of the study not according to inclusion criteria
45	Geoerger et al.	2017	Outcomes of the study not according to inclusion criteria
46	Schadendorf et al.	2017	Outcomes of the study not according to inclusion criteria
47	Yamazaki et al.	2017	Outcomes of the study not according to inclusion criteria
48	Kong et al.	2016	Patient population not according to inclusion criteria (Overlapping with other study)
49	Lebbe et al.	2014	Patient population and outcomes of the study not according to inclusion

Supplementary Table 4 : Studies referring to patients who discontinue ICI electively with a BOR PR/SD and relapse rates after ICI cessation.				
**Author, Year**	**Type of ICI use**	**Patients who discontinue electively on PR / SD [Total (PR / SD)]**	**Patients Relapse [Total (PR / SD)]**	**Relapse rate (n / N, %)**
Tang et al., 2024	Anti-PD1 monotherapy	8 (8 PR / 0 SD)	6 (6 PR/ 0 SD)	6 / 8 (75)
Rubatto et al., 2023	Anti-PD1 monotherapy	23 (17 PR / 6 SD)	5 (4 PR, 1 SD)	5 / 23 (21.7)
Ferdinandus et al., 2022	Comb. ICI, anti-PD1 monotherapy	16 (14 PR, 2 SD)	2 (2 PR, 0 SD)	2 / 16 (12.5)
Kartolo et al., 2022	Comb. ICI, anti-PD1 monotherapy	18 (13 PR, 5 SD)	NA	NA
Pokorny et al., 2021	Anti-PD1 monotherapy	39 (28 PR, 11 SD)	10 (7 PR, 3 SD)	10 / 39 (25.6)
Valentin et al., 2021	Anti-PD1 monotherapy	12 (12 PR or SD)	2 (PR or SD)	2 / 12 (13.3)
Warburton et al., 2020	Anti-PD1 monotherapy	9 (6 PR, 3 SD)	4 (2 PR, 2 SD)	4 / 9 (44.4)
Hamid et al., 2019	Anti-PD1 monotherapy	5 (5 PR)	1 (1 PR)	1 / 5 (20)
Jansen et al., 2019	Anti-PD1 monotherapy	60 (44 PR, 16 SD)	22 (14 PR, 8 SD)	22 / 60 (36.6)
Robert et al., 2019	Anti-PD1 monotherapy	82 (69 PR, 13 SD)	22 (16 PR, 6 SD)	22 / 82 (26.8)

Supp. Table 5: Studies referring to factors affecting time to progression after discontinuation and significant results of each study

Author, Year	Type of ICI use	Reason for discontinuation	Factors analyzed for the prediction of post-discontinuation PD	Significant factors
Chatzioannou et al., 2023	Combination ICI, Anti-PD1 monotherapy	Complete response	Type of immunotherapy (combination and single-agent), time to CR for therapy start, age	No statistically significant differences were detected regarding type of ICI, or time to CR after therapy start for time to PD. Age below 77 was associated with better PFS.
Ochenduszko et al., 2023	Anti-PD1 monotherapy	Complete response	Age, BMI, ECOG PS, time of anti-PD1 treatment, advanced disease stage, elevated LDH, baseline NLR >3, steroids or antibiotics use	Antibiotics use after discontinuation increased the risk for progression [OR 16.53 (95%CI 1.7 – 226.03)].
Rubatto et al., 2023	Anti-PD1 monotherapy	Complete response, TLT, patient/physician choice	**Total population:** Gender, ECOG status, LDH, anatomic location of the tumor, treatment duration (<6 or >6 months), BRAF status, Breslow, Clark level<br><br>**Complete responders:** primary tumor site and site of metastasis<br><br>**Toxicity:** Anatomic location, mutational status, type of response to 1st-line therapy	**Total population:** Mucosal subtypes and treatment duration before discontinuation <6 months led to increased risk for progression.<br><br>**Complete responders:** Mucosal lesions were associated with increased risk while metastasis limited to lung with decreased risk for progression<br><br>**Toxicity:** Unknown primary, NRAS mutational status, and PR after 1st-line therapy led to increased risk for progression
Ferdinandus et al., 2022	Combination ICI, Anti-PD1 monotherapy	Durable response, TLTs	Metabolic imaging, morphologic criteria on CT	Metabolic imaging predicted progression in a more accurate way compared to morphologic image, and patients with CMR demonstrated higher TTP after discontinuation compared to non-CMR (12.7 mo vs NR, P<0.001)
Kartolo et al., 2022	Combination ICI, Anti-PD1 monotherapy	Elective or TLTs	Reason for discontinuation	Not significant difference in OS between elective discontinuation of due to TLTs [19.7 vs 25.1 mo, p=0.991]. Similar results were drawn when the analysis was restricted to anti-PD1 inhibitors only.
Dimitriou et al., 2021	Combination ICI, Anti-PD1 monotherapy	CR or TLTs with CR	Reason for discontinuation	No significant different in time to PD among patients with CR compared to TLTs with CR.

Dutheil et al., 2021	type of ICI not defined	CR	Age, gender, tumor burden, anatomic location of metastasis, grade of toxicities, type of ICI, treatment duration, mutational status, histologic subtype and prior treatments	Wild type melanomas, mucosal, acral or unknown primary and prior treatment lines received were associated with increased risk for recurrence.
Gibney et al., 2021	Combination ICI, anti-PD1 monotherapy	Elective or TLTs	Gender, stage of disease, brain metastasis, ECOG PS, prior treatments, histologic subtype, type of treatment	Trend for shorter time to PD In patients with mucosal melanoma or patients treated with anti-CTL4/anti-PD1 (not significant differences).
Pokorny et al., 2021	Anti-PD1 monotherapy		Gender, age, pre-PD1 NLR, pre-PD1 LDH, post-PD1 NLR, post-PD1 LDH, BOR, brain metastasis	Younger age, history of brain metastasis and post-PD1 LDH were significant predictors of recurrence.
Schank et al. ,2021	Combination ICI, Anti-PD1 monotherapy	Elective or TLTs	Age, gender, mutational status, S100 baseline or on time of PET/CT, LDH baseline or on time of PET/CT, type of treatment, prior treatments, metabolic response	Patients with CMR demonstrated prolonged time to PD post discontinuation compared to non-CMR patients (p=0.007).
Van Zeijl et al., 2021	Anti-PD1 monotherapy	CR or elective	Time between start of anti-PD1 and PR/CR, first response to anti-PD1 discontinuation, status at discontinuation	Longer time from start of anti-PD1 to first reported PR or CR, PR at discontinuation were associated with increased risk for progression. CR / PR patients had statistically significant prolonged PFS post discontinuation compared to SD patients. Similar results for patients who discontinued electively, while for patients ceased for AEs, a statistically significant difference was not detected.
Valentin et al., 2021	Anti-PD1 monotherapy	CR, elective, TLTs	Age, gender, time on treatment, reason for discontinuation, mutational status, number of metastases, brain metastasis, LDH	No statistically significant factors were detected
Betof – Warner et al., 2020	Anti-PD1 monotherapy	CR	Time to CR	Time to CR was not significantly associated with risk of progression after CR (HR 0.94, p=0.16)
Mesnard et al., 2020	Anti-PD1 monotherapy	CR	Age, gender, ulceration, Breslow index, number of metastatic sites, brain metastasis, number and type of prior treatments, CMR on PET/CT	CMR on PET/CT was associated with decreased risk for relapse after discontinuation. Other analyzed parameters were not statistically significant.
Warburton et al., 2020	Anti-PD1 monotherapy	Disease control	ctDNA	Positive ctDNA after ICI cessation was associated with increased risk for relapse (RR 4, 95%CI 1.88 – 10.26), while undetectable ctDNA led to longer TFI.

Jansen et al., 2019	Anti-PD1 monotherapy	Disease control	Treatment duration to discontinuation, objective response at discontinuation, type of anti-PD1 used, mutational status, brain metastasis, number of metastases, LDH	CR at the time of discontinuation was associated with decreased risk for progression compared to non-CR patients (HR 0.27, 95%CI 0.13 – 0.54).
Robert et al., 2019	Anti-PD1 monotherapy	Disease control, elective	BOR during treatment, Duration of treatment	Patients with CR or PR had similar PFS rates at 24 months, while patients with SD progressed earlier. Contrary to, patients with CR who ceased treatment at 6 moths had similar PFS rates compared to patients who did complete 2 years of treatment.
Schvartsman et al., 2018	Anti-PD1 monotherapy	Elective or TLTs	Baseline characteristics	No baseline characteristics were associated significantly with relapse.

US: United States, TLTs: treatment-limiting toxicities, PD-1: programmed- cell death 1, PFS: progression-free survival, OS: overall survival, PD: progressive disease, NA: not available, DCR: disease control rate, CR: complete response, PR: partial response, SD: stable disease, ICI: immune checkpoint inhibitor, 95%CI: confidence interval, IQR: interquartile range, CMR: complete metabolic response, OR: odds ratio, NLR: neutrophil to leucocyte ratio, BMI: body mass index, TTP: time to progression, NR: not reached, TFI: treatment free interval,

**Supp. Table 6: Studies referring to PFS and OS of patients who discontinued ICIs for reason than progressive disease and significant factors of each study.**						
**Author, Year**	**Type of ICI use**	**Median time f.u. from treatment start**	**Median PFS**	**Median OS**	**Significant factors**	**Notes**
Fletcher et al., 2024	Anti-PD1 monotherapy	NA	34.4 mo	46.6 mo	Treatment duration did not significantly affect PFS and OS.	
Chatzioannou et al., 2023	Combination ICI, anti-PD1 monotherapy	22 (IQR 17-24)	NR (95% CI NR- NR)	NR (95% CI NR-NR)	Age below 77 years was associated with better PFS. Also, NR PFS and OS in CR patients, while lower survival times were reported in patients without non-CR.	Survival times are calculated from CR
Ochenduszko et al., 2023	anti-PD1 monotherapy	49.3 mo (95%CI 43.8 – 52.5)	NR (95%CI 62.3 – NR)	NR (95%CI 62.4 – NR)	-	
Sadrolashrafi et al., 2023	Combination ICI, anti-PD1 monotherapy	69.3 mo	25 mo (SD 34.7)	NR	-	
Walburton et al., 2023	Combination ICI	NA	NR	NR	Complete responders demonstrated higher PFS and OS compared to non-CR patients [PFS, HR 0.25, 95%CI 0.1 – 0.7 and OS, HR 0.32, 0.09 – 1.1]	
Ellebaek et al., 2022	Combination ICI, anti-PD1 monotherapy	NA	NA	NA	Improved OS rates in elective discontinuation group compared to TLTs (HR 0.19, 95%CI 0.07 – 0.53, p=0.013) and not significant PFS (HR 0.66, 95%CI 0.29 – 1.47, p=0.31)	
Kartolo et al.	Combination ICI, anti-PD1 monotherapy	NA	NA	NA	Neither number of metastasis at baseline, reason for discontinuation or CR/PR at time of discontinuation were associated with OS	From discontinuation
Asher et al., 2021	Combination ICI, anti-PD1 monotherapy	39.1 mo	NR (CR), 36.5 (PR), 12.8 (SD)	NR (CR), NR (PR), 24.6 (SD)	Statistically significant shorter PFS and OS in patients who did not achieve CR compared to CR patients. Higher risk for progression for patients	

					with grade 3-4 AEs compared to grade 0-2 or for patients who used steroids. Also, line of treatment of duration of treatment were significant predictors of PFS.	
Dimitriou et al., 2021	Combination ICI, anti-PD1 monotherapy	NA	NA	NR	Time to CR (<6 months vs >6 months) and reason for discontinuation not significantly associated with PFS.	From CR
Pokorny et al., 2021	anti-PD1 monotherapy	Available	NR	NA	-	-
Van Zeijl et al., 2021	anti-PD1 monotherapy	NA	NA	CR/ PR: NR<br>SD: 29 mo	Reason for discontinuation (elective or due to AEs) and BOR at discontinuation were significant predictors of OS.	
Betof-Warner et al., 2020	anti-PD1 monotherapy	NA	NR	NR	-	
Mesnard et al., 2020	anti-PD1 monotherapy	NA	20.5 (3-36)	NA	-	
Swami et al., 2020	Anti-PD1 monotherapy	30.3 (range 4.6 – 49.4)	24.6mo	NR	-	
Tikkanen et al., 2020	Anti-PD1 monotherapy	8 (range 0 – 44)	NA	38 mo	-	-
Walburton et al., 2020	Anti-PD1 monotherapy	NA	NR	NR	-	-
Bisschop et al., 2019	Anti-PD1 monotherapy	NA	NA	60% 24month rate	-	-
Robert et al., 2019	Anti-PD1 monotherapy	57.7 mo	NA	95.9% 24-month rate, 93.8% 36-month rate	-	

TLTs: treatment-limiting toxicities, PD-1: programmed- cell death 1, PFS: progression-free survival, OS: overall survival, PD: progressive disease, NA: not available, NR: not reached, CR: complete response, PR: partial response, SD: stable disease, ICI: immune checkpoint inhibitor, 95%CI: confidence interval, IQR: interquartile range, CMR: complete metabolic response, OR: odds ratio,, TTP: time to progression, TFI: treatment free interval,

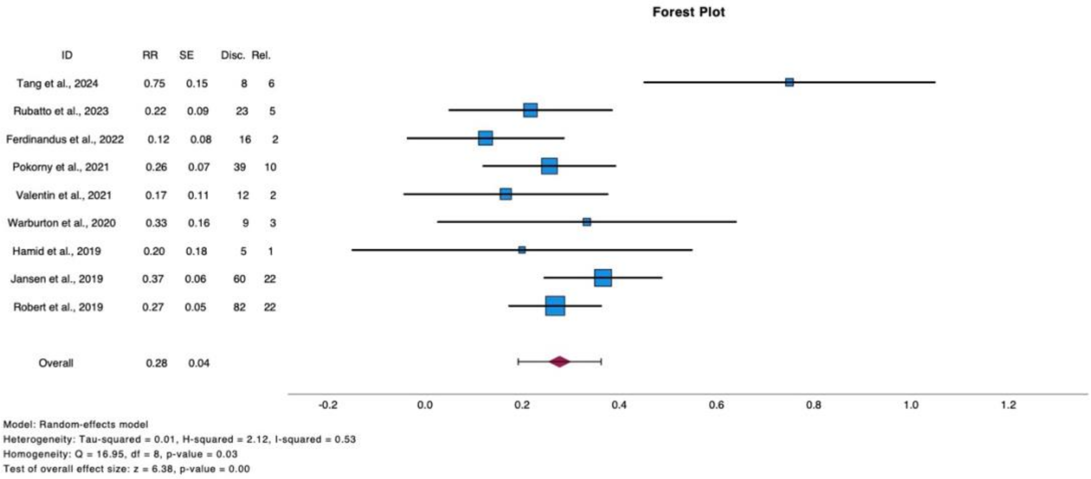

Supplementary. Figure 1: Forest plots demonstrate relapse rates (RR) in patients who discontinued treatment with ICI electively on PR/SD. (SE: standard error, Disc: patients who discontinue ICI, Rel: patients who relapsed after cessation).

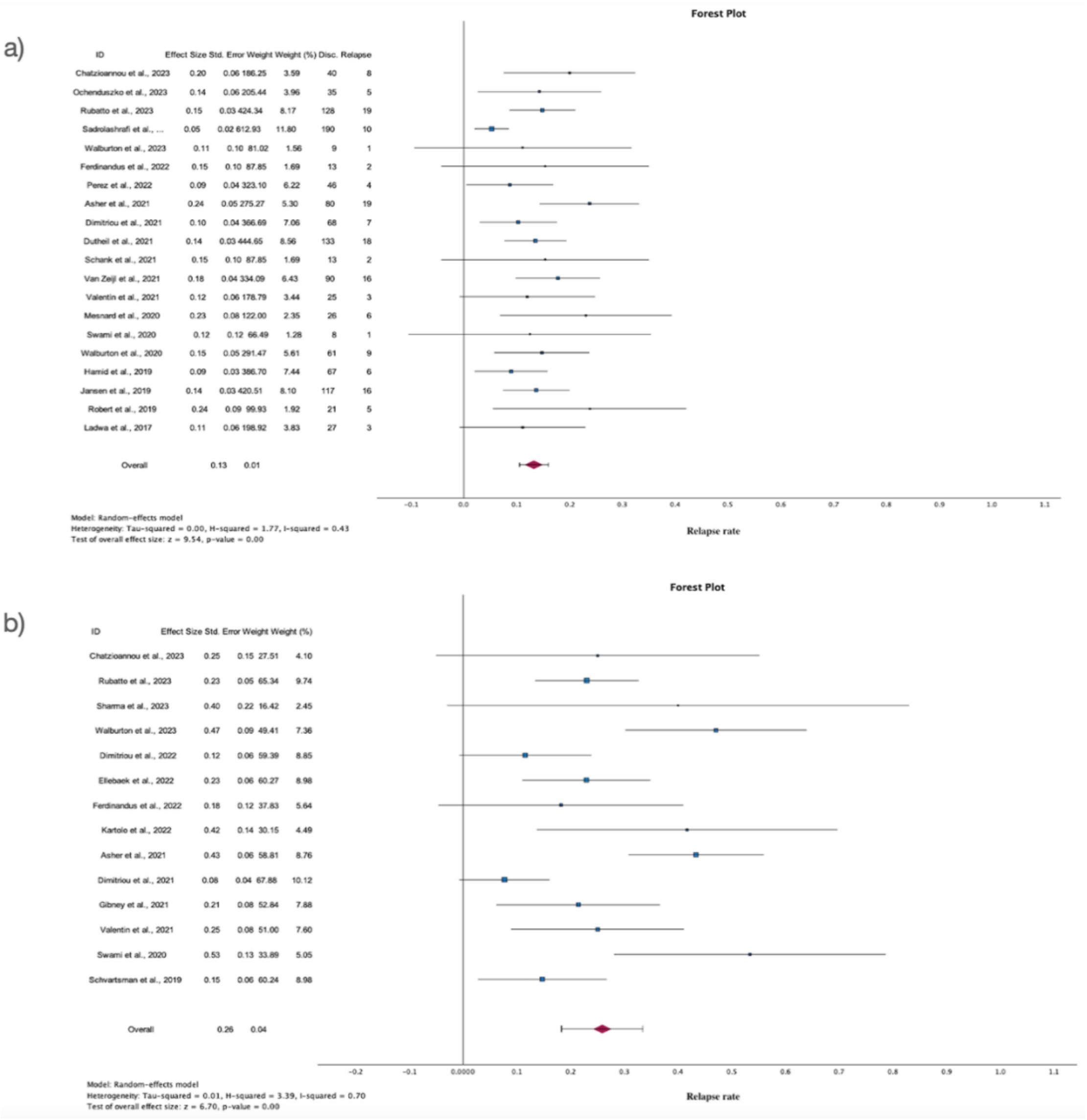

Supplementary. Figure 2: Forest plots demonstrate relapse rates in a) patients who discontinued treatment with ICI on CR and b) in patients who discontinue due to TLTs.

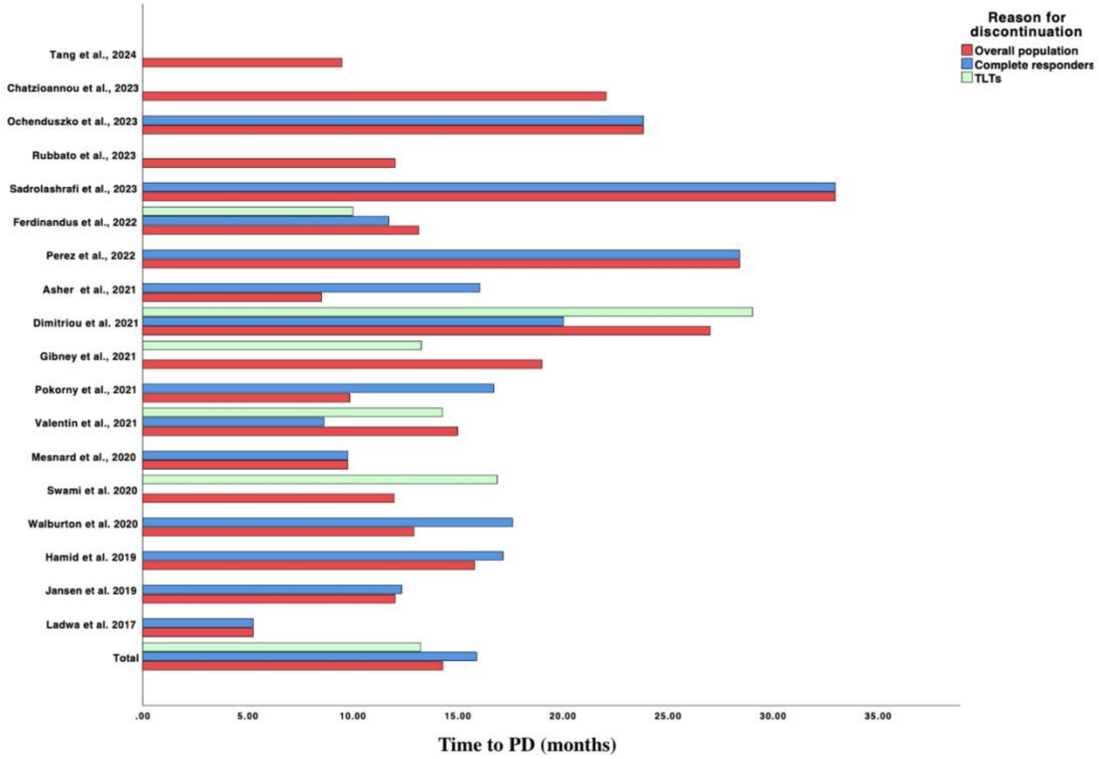

Supplementary Figure 3: Mean time to progressive disease in patients who discontinue treatment (red, overall population) and based on reason for discontinuation [complete response (blue color) and TLTs (light green color)].

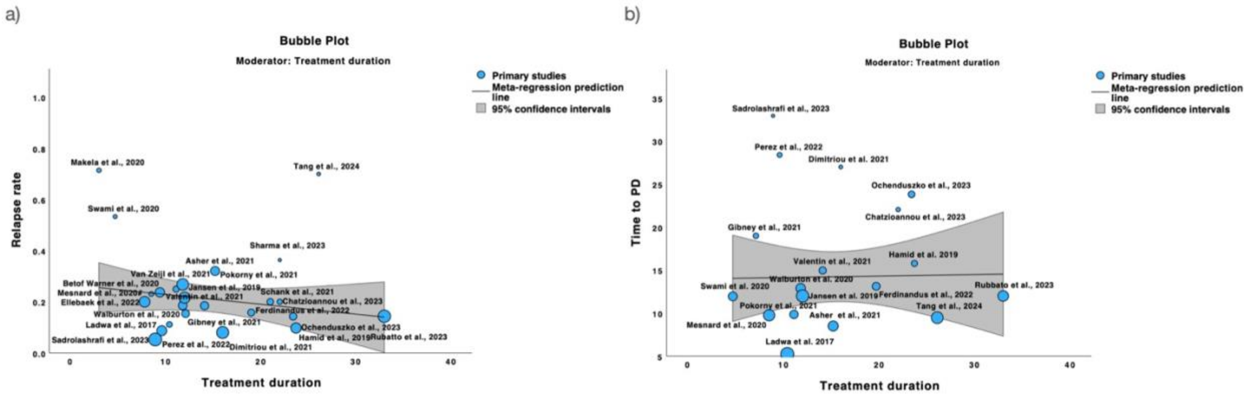

Supplementary Figure 4: Meta-regression analysis revealed not significant association between duration of treatment with ICI and a) risk of relapse (b=-0.004, p=0.273, 95%CI -0.11 to 0.03) or b) time to PD post cessation (b=0.017, p=0.925, 95%CI -0.36 to 0.39).

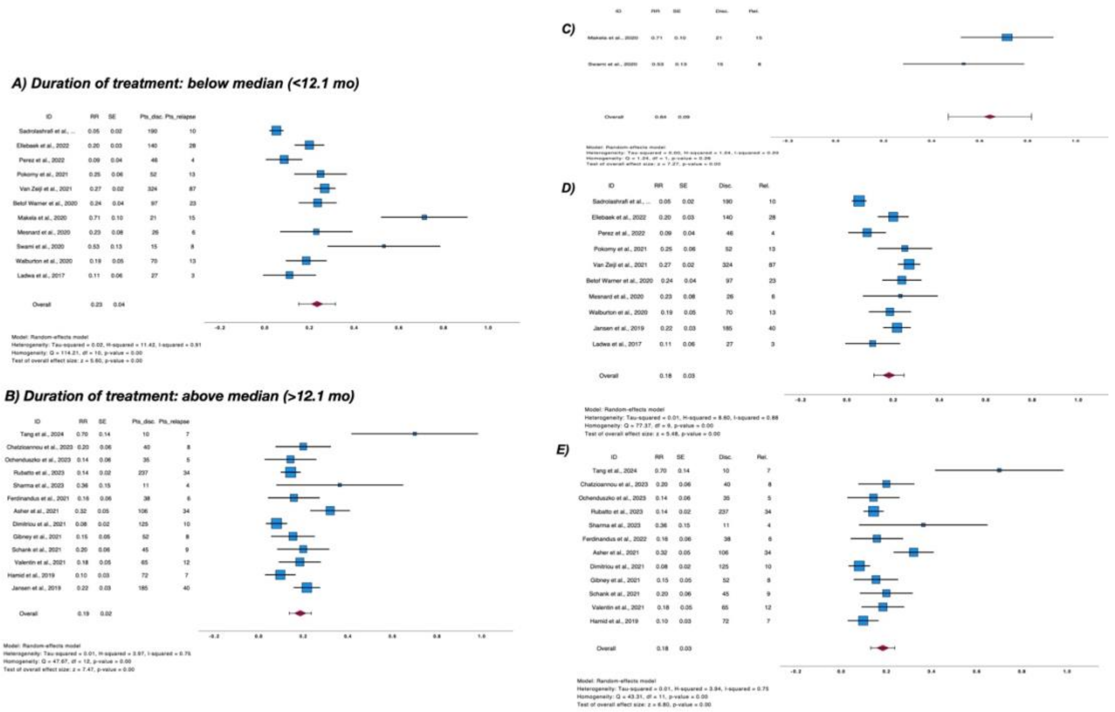

Supplementary Figure 5: Forest plots showing relapse rate in studies where treatment duration was a) below and b) above the median (12.1mo), and in studies where different thresholds of ICI treatment duration were used [c] 0-6 months, d) 6-12 months and e) >12 months].

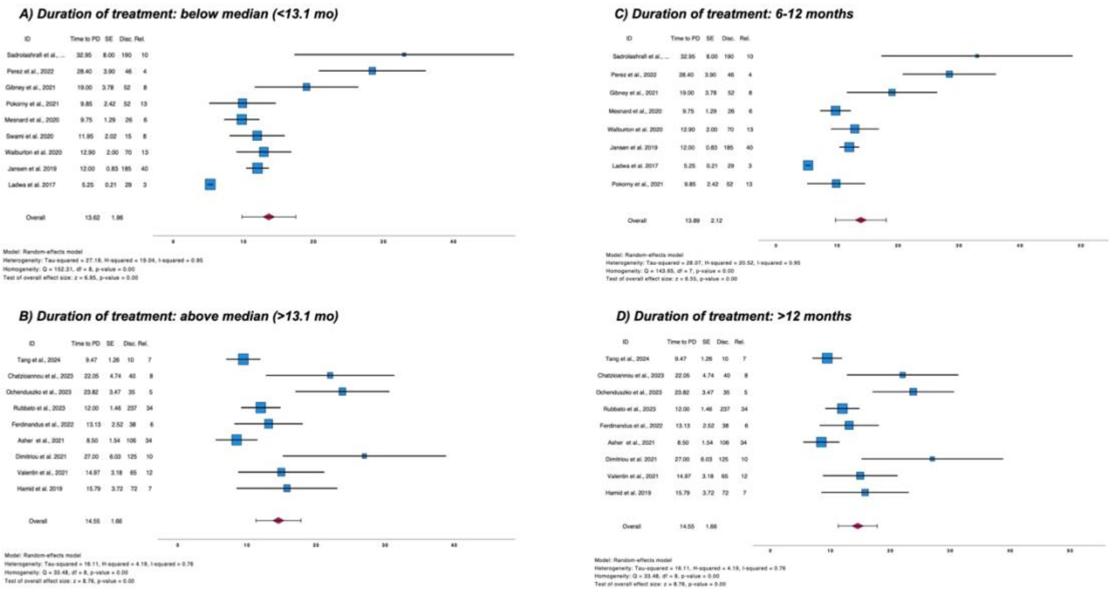

Supplementary Figure 6: Forest plot showing time to PD in studies where treatment duration was a) below (13.1mo) and, b) above median (13.1 mo), and in studies where different thresholds of ICI treatment duration were used [c]6-12 months and d) >12 months].

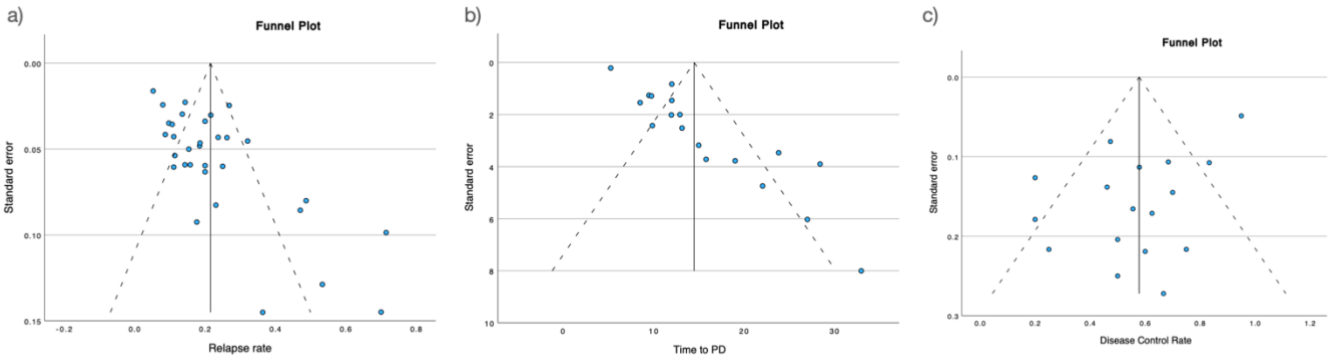

Supplementary Figure 7: Funnel plots assessing publication bias in a) relapse rate, b) time to PD, c) DCR after rechallenge.
